# Aberrant Membrane Composition and Biophysical Properties Impair Erythrocyte Morphology and Functionality in Elliptocytosis

**DOI:** 10.3390/biom10081120

**Published:** 2020-07-29

**Authors:** Hélène Pollet, Anne-Sophie Cloos, Amaury Stommen, Juliette Vanderroost, Louise Conrard, Adrien Paquot, Marine Ghodsi, Mélanie Carquin, Catherine Léonard, Manuel Guthmann, Maxime Lingurski, Christiane Vermylen, Theodore Killian, Laurent Gatto, Mark Rider, Sébastien Pyr dit Ruys, Didier Vertommen, Miikka Vikkula, Pascal Brouillard, Patrick Van Der Smissen, Giulio G. Muccioli, Donatienne Tyteca

**Affiliations:** 1 CELL Unit & PICT Imaging Platform, de Duve Institute, UCLouvain, 1200 Brussels, Belgium; hpollet@hotmail.com (H.P.); anne-sophie.cloos@uclouvain.be (A.-S.C.); amaury.stommen@uclouvain.be (A.S.); juliette.vanderroost@uclouvain.be (J.V.); louise.conrard.lc@gmail.com (L.C.); marine.ghodsi@student.uclouvain.be (M.G.); melanie.carquin@gmail.com (M.C.); c.leonard.hellin@gmail.com (C.L.); manuel.guthmann@student.uclouvain.be (M.G.); maxime.lingurski@uclouvain.be (M.L.); patrick.vandersmissen@uclouvain.be (P.V.D.S.); 2 Bioanalysis and Pharmacology of Bioactive Lipids Research Group, Louvain Drug Research Institute, UCLouvain, 1200 Brussels, Belgium; adrien.paquot@uclouvain.be (A.P.); giulio.muccioli@uclouvain.be (G.G.M.); 3 PEDI Unit, Institut de Recherche Expérimentale et Clinique & Saint-Luc Hospital, UCLouvain, 1200 Brussels, Belgium; christiane.vermylen@uclouvain.be; 4 Computational Biology and Bioinformatics Unit, de Duve Institute, UCLouvain, 1200 Brussels, Belgium; theodore.killian@uclouvain.be (T.K.); laurent.gatto@uclouvain.be (L.G.); 5 PHOS Unit & MASSPROT Proteomics Platform, de Duve Institute, UCLouvain, 1200 Brussels, Belgium; mark.rider@uclouvain.be (M.R.); sebastien.pyrditruys@uclouvain.be (S.P.d.R.); didier.vertommen@uclouvain.be (D.V.);; 6 Human Molecular Genetics, de Duve Institute, UCLouvain, 1200 Brussels, Belgium; miikka.vikkula@uclouvain.be (M.V.); pascal.brouillard@uclouvain.be (P.B.); 7 Walloon Excellence in Life Sciences and Biotechnology (WELBIO), de Duve Institute, UCLouvain, 1200 Brussels, Belgium

**Keywords:** spectrin cytoskeleton, Ca^2+^, lipid domains, membrane asymmetry, membrane rigidity, membrane curvature, lysophosphatidylserine, oxidative stress, amitriptyline

## Abstract

Red blood cell (RBC) deformability is altered in inherited RBC disorders but the mechanism behind this is poorly understood. Here, we explored the molecular, biophysical, morphological, and functional consequences of α-spectrin mutations in a patient with hereditary elliptocytosis (pEl) almost exclusively expressing the Pro260 variant of SPTA1 and her mother (pElm), heterozygous for this mutation. At the molecular level, the pEI RBC proteome was globally preserved but spectrin density at cell edges was increased. Decreased phosphatidylserine vs. increased lysophosphatidylserine species, and enhanced lipid peroxidation, methemoglobin, and plasma acid sphingomyelinase (aSMase) activity were observed. At the biophysical level, although membrane transversal asymmetry was preserved, curvature at RBC edges and rigidity were increased. Lipid domains were altered for membrane:cytoskeleton anchorage, cholesterol content and response to Ca^2+^ exchange stimulation. At the morphological and functional levels, pEl RBCs exhibited reduced size and circularity, increased fragility and impaired membrane Ca^2+^ exchanges. The contribution of increased membrane curvature to the pEl phenotype was shown by mechanistic experiments in healthy RBCs upon lysophosphatidylserine membrane insertion. The role of lipid domain defects was proved by cholesterol depletion and aSMase inhibition in pEl. The data indicate that aberrant membrane content and biophysical properties alter pEl RBC morphology and functionality.

## 1. Introduction

During its lifetime, the red blood cell (RBC) undergoes extensive deformations needed to pass through narrow capillaries to deliver oxygen to tissues. Such exceptional deformability relies on RBC intrinsic features, including a biconcave shape due to excess plasma membrane surface vs. cytoplasmic volume, a finely regulated cytoplasmic viscosity controlled by hemoglobin concentration, and a cytoskeleton composed of a meshwork of spectrin tetramers linked to the membrane by the 4.1R- and ankyrin-based anchorage complexes [1,2]. RBC deformation is also determined by the ion balance and subsequent volume control regulated by ion channels, symporters, antiporters, and pumps. Among ion channels, one can cite PIEZO1, a mechanosensitive non-selective cation channel recently identified as the link between mechanical forces, Ca^2+^ influx and RBC volume homeostasis. The Ca^2+^-activated K^+^ channel (named Gardos), the Cl^−^/HCO_3_^−^ antiporter Band3, and the plasma membrane Ca^2+^ ATPase pump (PMCA) are also essential for the RBC homeostasis. For instance, the transient increase in intracellular Ca^2^ activates Gardos channels, leading to cell dehydration and favoring local membrane:cytoskeleton uncoupling [3,4,5,6]. Finally, RBC deformation also depends on ATP content and antioxidant systems. 

RBC plasma membranes have a high cholesterol content compared to those of other cells [7] and show lipid clustering in domains. In the RBC outer membrane leaflet, three domains have been identified [7,8,9,10,11]. The first, associated with high-curvature membrane areas, is mainly enriched in cholesterol and gathers upon RBC deformation. The second and third domains, associated with low-curvature membrane areas, are enriched in ganglioside GM1, phosphatidylcholine and cholesterol (hereafter named GM1-enriched domains) and in sphingomyelin, phosphatidylcholine, and cholesterol (hereafter named sphingomyelin-enriched domains), respectively. GM1-enriched domain abundance increases upon PIEZO1 activation while sphingomyelin-enriched domain abundance increases with Ca^2+^ efflux [10,12]. A fourth type of domain, mainly enriched in ceramide, has been recently identified at the inner leaflet [13].

RBC deformability and lifespan are altered in inherited RBC disorders, leading to chronic hemolytic anemia [14,15]. Contrary to other hemolytic anemias, elliptocytosis is asymptomatic in 90% of cases, and so its prevalence is largely underestimated [16]. This heterogeneous disease affecting α-spectrin-, β-spectrin- or 4.1R-coding genes is inherited in an autosomal dominant manner, with rare cases of de novo mutations. The only treatments are folate therapy, transfusion, or splenectomy [17,18]. A recent simulation revealed that elliptocytotic RBCs are unable to recover their original biconcave shape after passing through splenic interendothelial slits due to impaired elasticity, leading to fragmentation when the RBC cytoskeleton connectivity is lower than 20% [19].

However, the relevance of this simulation remains to be tested in vitro and the molecular link between cytoskeleton alteration and RBC elasticity impairment is still unknown. In the present study, we took benefit from our expertise in the healthy RBC membrane analysis using complementary imaging, lipidomic and biophysical approaches to elucidate the mechanism behind morphology and functionality defects of RBCs in elliptocytosis. To this aim, we opted to study RBCs from patients with moderate to severe case of elliptocytosis, which is not so common since elliptocytosis is asymptomatic in 90% of cases. The patient who meets this criterion is a female teenager diagnosed with elliptocytosis during infancy (patient with elliptocytosis, pEl). She underwent cholecystectomy to eliminate cholelithiases accumulating in the gall bladder, but no splenectomy. None of the patient’s relatives were consulted for symptoms nor were they diagnosed with elliptocytosis, but her mother (mother of pEl, or pElm) nevertheless appeared mildly affected and her RBCs were used for comparison. Blood from healthy donors upon storage at 4 °C, known to be associated with RBC vesiculation, biochemical and morphology changes [13,20], was also used as internal control.

The pEl RBC membrane was compared to those of pElm and healthy donors at three levels. At the molecular level, we determined the membrane and cytoskeleton protein content, the extent of hemoglobin membrane interaction and its auto-oxidation, the membrane lipid content, the extent of lipid oxidation and the activity of the plasmatic acid sphingomyelinase (aSMase). At the biophysical level, we measured membrane curvature, rigidity, lateral heterogeneity in domains and transversal asymmetry. At the morphological and functional levels, we determined the RBC size and circularity, membrane Ca^2+^ exchanges using PDMS chambers, intracellular Ca^2+^ content and RBC fragility through hemoglobin release.

## 2. Materials and Methods 

### 2.1. Blood Collection and Preparation

The study was approved by the Medical Ethics Committee of UCLouvain. Blood was collected by venipuncture into K^+^/EDTA-coated tubes from 18 healthy volunteers, patient pEl and her mother pElm with written consent. Before experiments, blood was diluted 10-fold in glucose- and HEPES-containing medium (Invitrogen, Carlsbad, CA, USA). RBCs were then collected, washed twice by centrifugation at 200× *g* for 2 min and resuspended, as in [13]. The number of RBCs used for experiments were as follows: (i) imaging, 12.5 × 10^6^; (ii) phosphatidylserine (PS) surface exposure by flow cytometry, 0.5 × 10^6^; (iii) cholesterol assay, 37.5 × 10^6^; (iv) lipidomics, 250 × 10^6^; (v) Ca^2+^ and ROS contents, 15 × 10^6^; (vi) intracellular ATP, 5 × 10^6^; and (vii) hemoglobin release, 12.5 × 10^6^.

### 2.2. Chemical Treatment

All treatments, except amitriptyline, were performed on diluted washed RBCs. To activate PIEZO1, RBCs were incubated with 0.5 µM Yoda1 (Biotechne, Minneapolis, MD, USA) for 20 s at 22 °C. To inhibit voltage-dependent Ca^2+^ channels, RBCs were incubated with 1 µM ω-agatoxin (Alomone Labs, Jerusalem, Israel) for 20 min at 22 °C. To modulate the intracellular Ca^2+^, RBCs were preincubated either in a Ca^2+^-free medium containing 1 mM EGTA (Sigma-Aldrich) for 10 min at 22 °C or with 20–40 µM BAPTA-AM (Abcam) for 60–90 min at 37 °C. For cholesterol depletion, RBCs were preincubated with 0.9 mM methyl-β-cyclodextrin (mβCD; Sigma-Aldrich) for 30 min at 37 °C. To decrease oxidative stress, RBCs were preincubated with 1 mM ascorbic acid for 1 h at 37 °C. Lysophosphatidylserine (lysoPS; Sigma) was inserted into membranes at 3 µM in 0.01% (*w*/*v*) defatted bovine serum albumin (BSA; Sigma)-containing medium for 5 or 40 min at 37 °C and was maintained during the whole experiment. For plasma acid sphingomyelinase (aSMase) inhibition, whole blood was incubated with 5 µM amitriptyline (Sigma-Aldrich, Saint-Louis, MO, USA) for 1 h at 37 °C, then diluted and washed as above.

### 2.3. Membrane Lateral Heterogeneity, Curvature and Transversal Asymmetry’

Endogenous cholesterol labeling by mCherry-Theta toxin fragment, BODIPY-lipid membrane insertion and co-labeling were performed as in [10]. BODIPY-ceramide-labeled RBCs were submitted to surface back-exchange at 22 °C with 5% (*w*/*v*) BSA (twice 10 min). All poly-L-lysine (PLL)-coated coverslips were then laid down in labTek chambers and immediately observed with a Zeiss LSM510 confocal microscope (plan-Apochromat 63X 1.4 oil objective) or a wide-field fluorescence microscope (Observer.Z1; plan-Apochromat 100X 1.4 oil Ph3 objective). For membrane curvature, the last version of Shape Analysis by Fourier Descriptors computation plugging for ImageJ was used as described in [12]. Membrane transversal asymmetry was assessed by PS externalization using Annexin-V FITC (Invitrogen), as in [13].

### 2.4. Lipid Quantification

Cholesterol content and lipid peroxidation were measured using Amplex Red cholesterol (Invitrogen) [9] and malondialdehyde (Abcam, Cambridge, United Kingdom) [21] kits, respectively. Malondialdehyde levels were expressed relative to hemoglobin content and cholesterol levels were expressed relative to total phospholipid content evaluated by phosphorus assay after lipid extraction. aSMase activity was measured on plasma isolated after ficoll separation using a SMase kit (Abcam). Phospholipids, lysophospholipids, sphingomyelin, sphingosine, ceramide and oxysterols were quantified by liquid chromatography–mass spectrometry as previously [13,22,23].

### 2.5. Ca^2+^, ATP, and ROS Measurements

Ca^2+^ content and exchanges were measured by Fluo-4 AM [12], a probe which minimizes interference with hemoglobin as compared with other Ca^2+^-specific probes but only generates qualitative measurements [24]. Intracellular and extracellular ATP levels were measured using a chemiluminescence assay kit (Abcam) as in [10,13]. Intracellular ROS content was determined in RBCs incubated in suspension with 15 µM 2′,7′-dichlorodihydrofluorescein diacetate (H_2_DCFDA; Invitrogen) in Krebs-Ringer medium for 60 min at 37 °C. RBCs were then pelleted and resuspended for fluorescence measurements (GloMax; Promega) at λ_exc_ of 490 nm. Data were expressed relative to the RBC mean corpuscular volume (MCV).

### 2.6. Scanning Electron Microscopy of RBCs on Filters

Washed RBCs were prepared, mounted and observed in the CM12 electron microscope with the SED detector at 80 kV, as in [13].

### 2.7. Immunofluorescence Staining of RBC Membrane and Cytoskeleton Proteins

Diluted washed RBCs were spread onto PLL-coated coverslips for 4 min, fixed with 4% (*v*/*v*) paraformaldehyde for 10 min and blocked with 1% (*w*/*v*) BSA for 30 min. To label membrane proteins, RBCs were then incubated for 1 h with rabbit monoclonal antibodies to glycophorin C (GPC) together with mouse monoclonal antibodies to CD47 (Invitrogen), washed 4 times in 1% (*w*/*v*) BSA, incubated for 1 h with the appropriate Alexa-secondary antibodies (5 µg/mL) and washed 4 times with 1% BSA. Total spectrin was revealed with antibodies against α/β-spectrins (Abcam) using the same protocol as above except that a permeabilization step with 0.5% (*w*/*v*) Triton X-100 for 3 min was done before the fixation. All coverslips were mounted in Mowiol in the dark for 24 h and examined with a Zeiss LSM510 confocal microscope using a plan-Apochromat 63× NA 1.4 oil immersion objective.

### 2.8. RBC Hemoglobin Release Measurements

Three types of measurements were performed. First, to evaluate the effect of a chemical agent, washed RBCs were incubated with the molecule in isosmotic medium (i.e., 320 mOsm) and pelleted by centrifugation at 200× *g* for 2 min. Supernatants and pellets broken with 0.2% (*w*/*v*) Triton X-100 were both assessed for hemoglobin at 450 nm in 96-well plates (SpectraCountTM, Packard BioScience Co, San Diego, CA, USA). Hemoglobin release in the supernatant was expressed as percentage of the total hemoglobin present in the sample. Second, to compare RBC fragility between donors, washed RBCs were incubated in isosmotic medium for 1 h at 37 °C under constant agitation, centrifuged, collected and measured for hemoglobin release as above. Third, cryohemolysis was measured at Saint-Luc Hospital following Strechman and Gescheidt’s protocol [25].

### 2.9. Calpain Activity

RBCs were lysed in the buffer provided in the Calpain activity assay kit (Abcam). To determine the calpain activity, 25 µg of proteins in the RBC lysates were mixed with the “reaction buffer” and the calpain substrate, incubated for 1 h at 37 °C and measured for fluorescence, as in [13].

### 2.10. Methemoglobin Determination

RBC lysates obtained through repeated freeze-thaw cycles were analyzed for methemoglobin content following indications of a sandwich Elisa assay kit (LifeSpan Biosciences, Seattle, WA, USA). Methemoglobin was finally detected by spectrophotometry (SpectraCount, Packard, San Diego, CA, USA) at 450 nm, as in [13].

### 2.11. Image Analysis and Data Quantification

RBC morphology (area, perimeter, circularity), spectrin intensity/occupation and line intensity profiles on confocal images as well as hemoglobin abundance on SDS-PAGE were determined using the Fiji software. Line intensity profiles were further analyzed for the (non)-overlapping between GPC and CD47 as follows. After threshold value determination to define the effective dynamic range, peaks were identified and classified into three categories: (i) only red, indicating non-overlapping of GPC with CD47; (ii) only green, indicating non-overlapping of CD47 with GPC; and (iii) red + green, indicating overlapping between both proteins. The abundance of peaks in each category was then expressed as percentage of total peaks. Lipid domain abundance/hemi-RBC surface was assessed by manual counting on confocal or fluorescence images and expressed by reference to the hemi-RBC projected area.

### 2.12. Data Presentation and Statistical Analyses

Data are expressed as means ± SEM when the number of independent experiments was *n* ≥ 3 or means ± SD if *n* ≤ 2, except for RBC circularity. In the latter case, a representative experiment is presented in the figure to highlight the distribution within the sample while the statistical analysis is carried out on means of all independent experiments and is indicated in the figure legend.

Statistical tests were performed only when *n* ≥ 3. First, for microscopy experiments (i.e., lipid domain abundance, RBC morphology, spectrin, and GPC/CD47 immunolabeling), unpaired *t*-test or one-way ANOVA followed by Turkey’s multiple comparison tests were used. In the other cases, non-parametrical tests (Mann–Whitney test or Kruskal–Wallis followed by Dunn’s comparison test) were preferred. Second, to evaluate the effect of chemical agents, the paired data were analyzed by: (i) paired t tests (lipid domains and membrane curvature); (ii) Wilcoxon matched-pairs signed rank tests (biochemical experiments); or (iii) unpaired t test (RBC circularity). ns, not significant; *, *p* < 0.05; **, *p* < 0.01; ***, *p* < 0.001, ****, *p* < 0.0001

Quantitative proteomics data were generated with ProteomeDiscoverer (ThermoFisher) and differential expression between control and pEI triplicates was statistically assessed using linear models and the empirical Bayesian model, as implemented in the Bioconductor [26] limma [27] package. After correction for multiple testing, proteins with adjusted *p*-values < 0.05 were deemed deferentially expressed. Pathway Studio (2019–2020, Elsevier Life Sciences, Amsterdam, The Netherlands) was used to determine enrichment pathways from proteomics data.

## 3. Results

### 3.1. pEl Presented a Severe to Moderate Elliptocytosis

**Figure 1:**
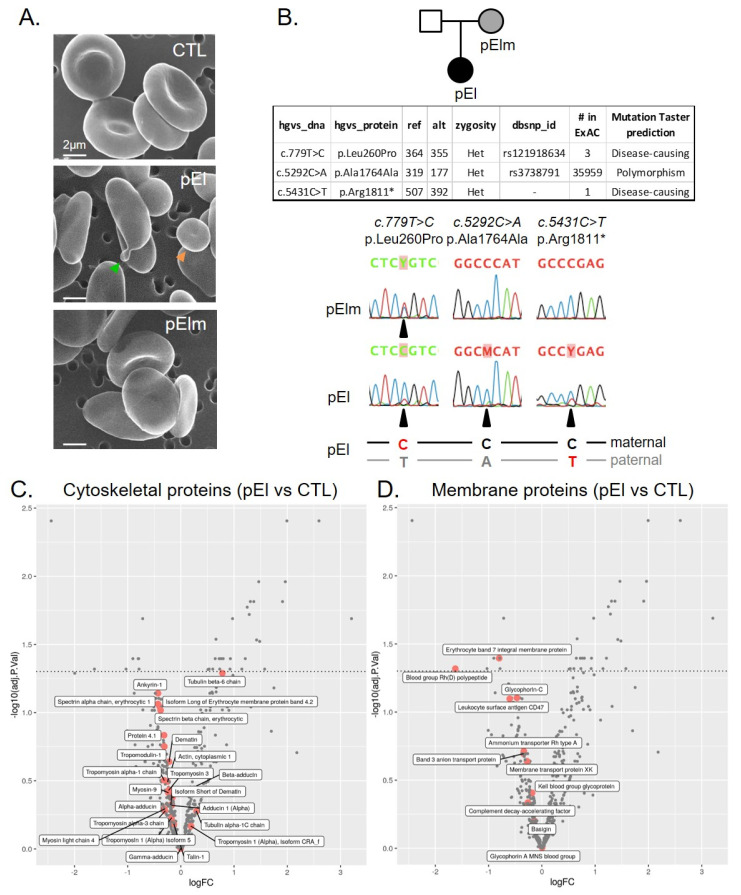
Patient pEl expressed predominantly the Pro260 variant of SPTA1 and exhibited RBCs with an elliptic shape but a globally preserved cytoskeletal and membrane protein content. (**A**) RBC morphology evaluated on RBCs fixed in suspension, laid down on filters and analyzed by scanning electron microscopy. Images are representative of 2 independent experiments. Orange arrowhead, small spherical RBC; green arrowhead, poïkilocytotic RBC. (**B**) Pedigree and genetic defects in patient pEl. pElm was partially affected but not diagnosed. Table. NGS results for patient pEl showing coverage (ref, reads with reference allele; alt; alternative allele), zygosity, code in dbSNP, number of occurrences in control population (The Exome Aggregation Consortium) and impact prediction from Mutation Taster, for two likely mutations and one heterozygous polymorphism in *SPTA1*. Sequences. mRNA sequencing showing partial loss of the allele carrying the premature Stop codon (T-allele at position 5431) in patient pEl. Maternal pElm is heterozygous for Leu260 Pro, whereas patient pEl is almost homozygous for the C at position 779 (Proline) and at position 5292, due to degradation. Y = T or C; M = C or A. (**C**,**D**) Cytoskeletal (**C**) and membrane (**D**) protein contents evaluated by differential quantitative mass spectrometry on ghost membranes. Volcanoplots show the log_2_ fold changes (logFC) in patient pEl vs. a healthy donor from 3 independent ghost preparations. Proteins showing a negative or a positive logFC are decreased or increased in patient pEl vs. the healthy donor, respectively. Proteins above the dotted line show a significant difference (*p* < 0.05) in patient pEl as compared to the healthy donor.

pEl blood sample analyses revealed enhanced bilirubin, lower hemoglobin, higher reticulocyte count, lower RBC mean corpuscular volume (MCV) and mean corpuscular hemoglobin (MCH) and elevated mean corpuscular hemoglobin concentration (MCHC), despite variation throughout the study. RBC abundance was less affected (Figure S1A–G). RBC distribution width and scanning electron microscopy revealed anisocytosis dominated by small elliptic cells and few poikilocytes (Figure 1A and Figure S1H). Decreased size and circularity of pEl RBCs were confirmed on blood smears and on poly-L-lysine-coated coverslips (Figures S1I,J and S2A,B) and did not result from the patient young age (Figure S2C–E). Maternal pElm RBCs also displayed a larger distribution of circularity, which might suggest the presence of some elliptic cells, as for pEl, but with no RBC surface loss (Figure 1A and Figure S2A,B). All those changes in pEl, in particular the high number of small RBCs [28], suggested that the patient was affected by a severe elliptocytosis during infancy and then appeared to have compensated anemia of moderate severity.

### 3.2. pEl Predominantly Expressed the Pro260 Variant of SPTA1

Next-Generation sequencing of pEl unraveled two heterozygous nucleotide substitutions in *SPTA1*, the gene encoding α-spectrin, resulting in a leucine-to-proline mutation (*c.779T* > *C*; p.Leu260Pro) and a premature stop codon (*c.5431C* > *T*; p.Arg1811*) (table from Figure 1B). The *c.779T* > *C* mutation was inherited from the mother, pElm, as confirmed by direct RNA sequencing (Figure 1B, bottom left). In pEl, the C-allele (corresponding to proline) was predominant at position c.779, with almost complete loss of the T-allele (corresponding to leucine). This is likely due to degradation by nonsense-mediated mRNA decay of the RNA of the paternal allele carrying the premature stop codon *c.5431C* > *T* (Figure 1B, bottom right). A reduced quantity of the paternal mRNA allele was indeed confirmed by sequencing a common polymorphism, which was heterozygous in pEl DNA (*c.5292C* > A; p.Ala1764Ala; table from Figure 1B) but skewed in its RNA (Figure 1B, bottom center). The phase of the alleles was deduced (Figure 1B, bottom), showing that pEl expressed mainly the proline-mutated allele inherited from her mother. In contrast, her second allele, carrying the premature stop codon (p.Arg1811*), as well as the A-allele of the polymorphism at position *c.5292*, was degraded. Thus, at the protein level, pEl should almost exclusively express the Pro260 variant of SPTA1. This Pro260 variant is known to exhibit reduced spectrin tetramer formation [29,30,31]. However, consequences for RBC morphology and functionality were not evaluated.

### 3.3. pEI RBCs Had Higher Spectrin Density at RBC Edges and Lower Segregation Between the Two Membrane Anchorage Complexes

**Figure 2:**
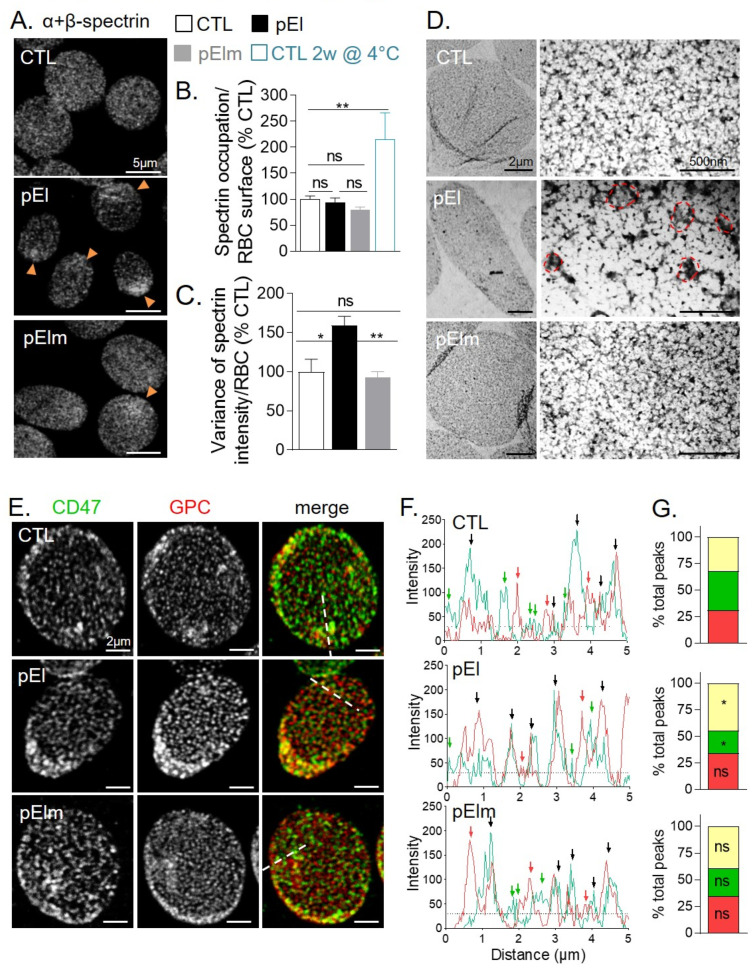
The spectrin network in patient pEl RBCs was heterogeneously distributed and the two membrane anchorage complexes were less segregated than in RBCs from healthy individuals and pElm. (**A**–**D**) Spectrin network analyzed by confocal (**A**–**C**) and transmission electron microscopy (**D**). RBCs were laid down on poly-L-lysine (PLL)-precoated coverslips (**A**–**C**) or formvar coated grids (**D**), permeabilized with 0.5% (*w*/*v*) Triton X-100, fixed and stained with anti-pan spectrin antibodies (**A**–**C**) or not (**D**). (**A**,**D**) Representative images. Orange arrowheads at A and red areas at D point to increased cytoskeleton density. (**B**,**C**) Quantification of spectrin occupation and dispersion on confocal images. 2 week-stored RBCs were used as internal control. Data are expressed by reference to the mean of control RBCs from the same experiment and are means ± SEM from 3–5 independent experiments with 50–150 RBCs measured/condition. Ordinary one-way Anova followed by Turkey’s multiple comparisons test. (**E**–**G**) Membrane protein distribution determined by confocal imaging. RBCs were laid down on PLL-precoated coverslips, fixed and stained with antibodies against CD47 (ankyrin complexes; green) and glycophorin C (GPC; 4.1R complexes; red). (**E**) Representative images. (**F**) Representative intensity profiles along the paths indicated at E by white dotted lines. Green arrows, CD47 only; red arrows, GPC only; black arrows, CD47/GPC overlapping. (**G**) Quantification of the abundance of CD47, GPC and CD47/GPC-overlapping peaks (yellow portion of the columns) determined on 30–40 profiles per condition generated from 4–5 independent experiments (expressed as percentage of total peaks from all the profiles). Unpaired *t*-tests were used to compare the proportion of yellow, green and red peaks between the CTL and the patient or her mother. ns, not significant; *, *p* < 0.05; **, *p* < 0.01.

Relative quantification of cytoskeletal and membrane proteomic profiles revealed no obvious modification in pEl RBCs, except a slight decrease in α- and β-spectrin, ankyrin-1 and Band3, a significant increase in tubulin β6 and a significant decrease in blood group Rh(D) polypeptide and Band7 (Figure 1C,D). Accordingly, SDS-PAGE/western blotting evidenced that the α- and β-spectrin-to-Band3 ratios were not modified in pEl vs. healthy donors whereas the Band7-to-Band3 was decreased by ~30% (data not shown). Those data indicated that protein modifications in pEl did not result from the higher reticulocyte content since reticulocyte membranes instead show higher β-spectrin- and Band7-to-Band3 ratio as compared to mature RBCs [32]. The cytoskeleton:membrane occupancy was also preserved in pEl RBCs, in contrast to stored RBCs used as positive control, but its distribution was more heterogeneous (Figure 2A–C and Figure S3A), showing spectrin gathering at one cell edge in ~75% of pEl RBCs vs. ~20% in pElm RBCs and ~5% in healthy RBCs (data not shown). Spectrin gathering was mainly seen in RBCs with elliptic but not circular shapes (Figure S3B). Moreover, at the nanoscale level, areas of reduced density alternating with darker areas were revealed in pEl RBCs (Figure 2D), suggesting the reorganization of cytoskeleton:membrane anchorage complexes. Accordingly, the spatial dissociation between CD47 and GPC respectively enriched in the ankyrin- and 4.1R- based complexes, was decreased in pEl RBCs but not in pElm RBCs (Figure 2E–G and Figure S4).

### 3.4. pEI RBCs Had Increased Membrane Curvature and Rigidity but Preserved Transversal Asymmetry

**Figure 3:**
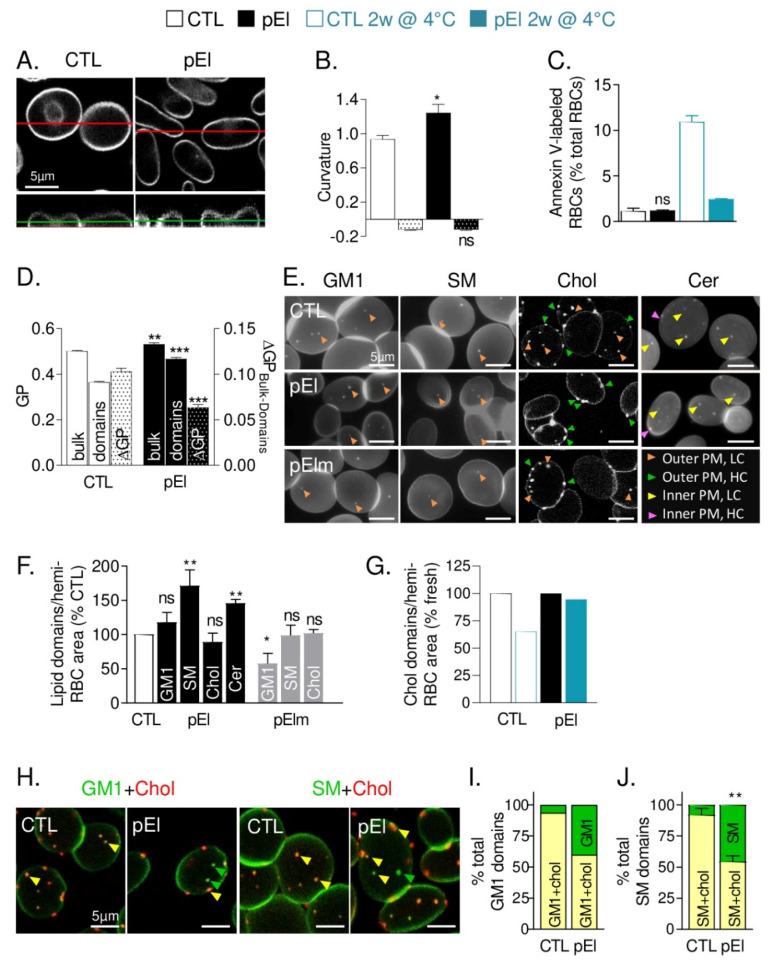
The RBC membrane from patient pEl exhibited increased curvature in high curvature areas, enhanced rigidity, impaired low curvature-associated lipid domains but preserved transversal asymmetry. (**A**,**B**) Membrane curvature. (**A**) RBCs were labeled with BODIPY-GM1, laid down in IBIDI chambers and analyzed by vital confocal microscopy. The figure shows in upper panels X-Y sections (positions indicated by the green lines in the lower panels) and in lower panels the corresponding X-Z sections (positions indicated by the red lines in the upper panels). Images are representative of 2 independent experiments. (**B**) RBCs were laid down in IBIDI chambers and analyzed in side view for membrane curvature profiles. The highest and lowest curvatures of each membrane curvature profile were then determined (open and dotted columns, respectively). Data are representative of 5 independent experiments with 6–16 RBCs analyzed per experiment. Unpaired t tests to compare CTL vs. pEl in high curvature areas and CTL vs. pEl in low curvature areas. (**C**) Membrane asymmetry. Surface exposition of phosphatidylserine (PS) evaluated by flow cytometry with Annexin V-FITC on fresh or 2-week-old RBCs. Data are means ± SD/SEM of 2–3 independent experiments Mann-Whitney test. (**D**) Membrane and domain lipid order. RBCs were labeled with Laurdan, spread on PLL-coated coverslips, observed by vital multiphoton microscopy and quantified for bulk and domain generalized polarization (GP) values (left axis) and difference in GP values between bulk membrane and domains (∆GP_bulk-domains_, dotted columns, right axis). Results are adapted from [33]. (**E**,**F**) Abundance of lipid domains on fresh RBCs. RBCs were either spread onto PLL-coated coverslips and labeled with BODIPY-GM1, -sphingomyelin (SM) or -ceramide (Cer) or labeled in suspension with mCherry-Theta (to detect endogenous cholesterol; chol) and spread onto coverslips. All coverslips were directly visualized by vital fluorescence/confocal microscopy. (**E**) Representative images. Green and pink arrowheads, outer and inner plasma membrane (PM) domains in high curvature (HC) respectively; orange and yellow arrowheads, outer and inner leaflet PM domains in low curvature areas (LC) respectively. (**F**) Quantification of lipid domain abundance per hemi-RBC area. Data are means ± SEM from 3–17 independent experiments/lipid in which 200–600 RBCs were counted/experiment. Unpaired t tests with Welch’s correction to compare abundance of lipid domains between the CTL and the patient or her mother. (**G**) Abundance of cholesterol-enriched domains on RBCs upon blood storage at 4 °C. RBCs were stored at 4 °C for 2 weeks, labeled for mCherry-Theta and processed as in E,F. Quantification of lipid domain abundance per hemi-RBC area. Data are means ± SD of 2 independent experiments in which 548–961 RBCs were counted/condition. (**H**–**J**) Spatial association of GM1- or sphingomyelin-enriched domains with cholesterol-enriched domains in low RBC curvature areas. RBCs were labeled in suspension with mCherry-Theta, spread onto PLL-coated coverslips and labeled with BODIPY-GM1 or -sphingomyelin. (**H**) Representative images. Green arrowheads, BODIPY-GM1 or -sphingomyelin (SM) only; yellow arrowheads, BODIPY-GM1/Theta or BODIPY-sphingomyelin/Theta co-labeled domains. For single channel images, see Figure S7A. (**I**,**J**) Quantification of the spatial association (yellow parts of the columns). Data are means ± SD/SEM of 2 and 3 independent experiments in which 50–100 RBCs were counted. Unpaired t test with Welch’s correction. ns, not significant; *, *p* < 0.05; **, *p* < 0.01; ***, *p* < 0.001.

We next examined whether defects in spectrin and membrane protein distribution could in turn modify membrane biophysical properties. pEl RBCs exhibited increased curvature at cell edges (Figure 3A,B). In contrast, membrane asymmetry, assessed by PS surface exposure, was unchanged in pEl RBCs, even upon storage (Figure 3C). The bulk membrane and domain lipid order, evaluated by generalized polarization (GP) of Laurdan, both increased in pEl RBCs and led to decreased ∆GP_bulk-domains_ (Figure 3D reproduced from [33,34]). As expected based on our previous work [33], this resulted in reduced release of extracellular vesicles both in fresh and stored blood samples of pEl vs. healthy donors (Figure S5A–C). Spherocyte abundance and RBC projected area were also less rapidly modified in pEl RBCs than in controls upon storage at 4 °C but the spectrin gathering was exacerbated (Figure S5D–F). This suggested no loss of pEl RBC membranes by microvesiculation or fragmentation but rather membrane reorganization. The higher content of components of the protein biosynthesis/folding machinery in pEl RBCs (Figure S6A) suggested that this reorganization already occurred during erythropoiesis.

### 3.5. Low Curvature-Associated Lipid Domains of pEI RBCs Exhibited Altered Abundance, Cholesterol Content and Membrane:Cytoskeleton Anchorage

To explore the potential membrane reorganization in pEl RBCs, we examined lipid domain abundance and surface distribution. In the RBC low curvature areas (i.e., the center of spread RBCs [12]), sphingomyelin-, but not GM1-, enriched domains significantly increased in pEl but not in pElm RBCs (Figure 3E,F). This increase did not result from the patient young age (Figure S2F). In contrast, cholesterol-enriched domains, associated with both high- and low-curvature areas (i.e., RBC periphery and center [12]) in healthy RBCs, were almost absent from low-curvature areas in pEl RBCs (Figure 3E). This was not due to loss by vesiculation, since total domain abundance was not decreased either in fresh or in stored pEl RBCs (Figure 3F,G). This observation suggested higher dissociation between GM1- or sphingomyelin- and cholesterol-enriched domains in pEl than in control RBCs, as confirmed by double-labeling (Figure 3H–J and Figure S7A). An increase in ceramide-enriched domains was also observed in pEl RBCs labelled with BODIPY-ceramide (Figure 3F). Membrane-inserted BODIPY-ceramide was resistant to BSA back-exchange in pEl RBCs (Figure S7B), suggesting ceramide-enriched domain association with the inner membrane leaflet as in healthy RBCs [13]. In addition to altered lipid domain abundance and surface distribution, we also showed that sphingomyelin-enriched domains in pEl RBCs were less stable in time and space, suggesting impaired cytoskeleton anchorage (Figure S7C). In conclusion, those data indicated that low curvature-associated lipid domains in pEl RBCs were altered in abundance, cholesterol content, and membrane:cytoskeleton anchorage. 

### 3.6. pEI RBCs Displayed Reduced Level of Phosphatidylcholine and PS Species But Increased lysoPS and Cholesterol Content

**Figure 4:**
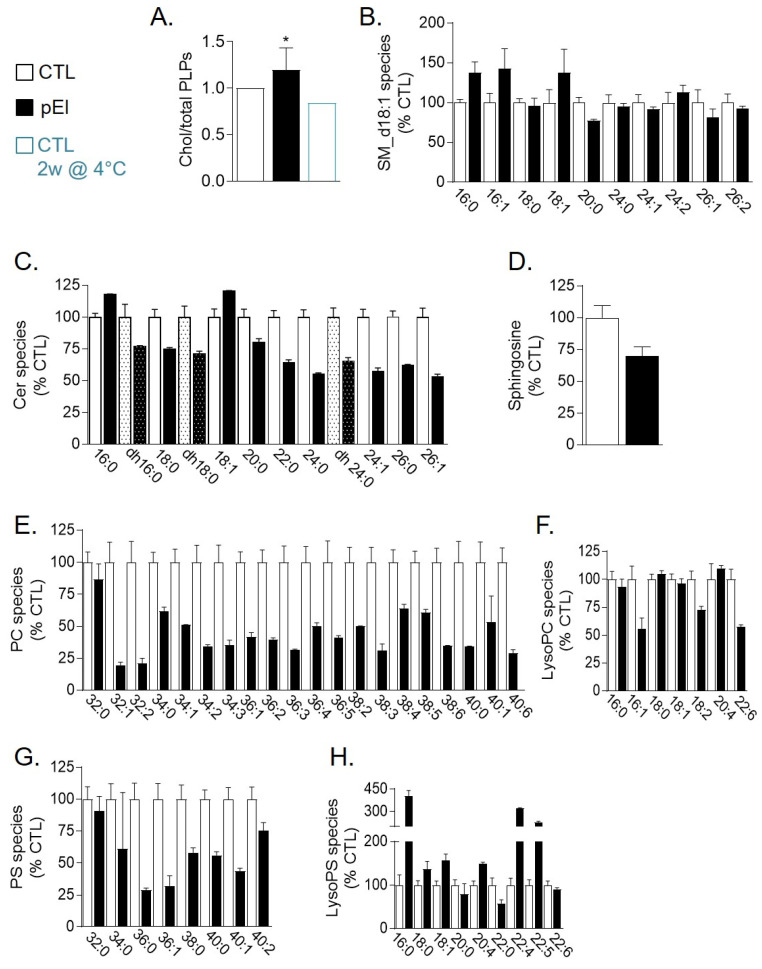
RBC membranes from patient pEI presented a lower abundance of phosphatidylcholine and phosphatidylserine species vs. an increased content in cholesterol and lysophosphatidylserine species. (**A**) Cholesterol content. RBCs were washed, lysed and assessed for cholesterol (chol) and total phospholipids (PLPs). Results are expressed as cholesterol/PLP ratio (chol/PLPs). Data are means ± SEM of 4 independent experiments, each in triplicate (except for blood upon storage at 4 °C). Mann-Whitney test. (**B**,**E**–**H**) Content in sphingomyelin (d18:1 D-erythro-sphingosine backbone), phosphatidylcholine (PC), lysophosphatidylcholine (lysoPC), phosphatidylserine (PS) and lysophosphatidylserine (lysoPS) species. RBCs were washed, lysed, extracted for lipids and assessed by lipidomics. (**C**,**D**) Ceramide, dihydroceramide (dotted columns) and sphingosine species assessed by HPLC-MS on washed, lysed and lipid-extracted RBCs. Species are classified based on fatty acid length and unsaturation number and results are expressed as percentage of controls (CTL, mean of 9 healthy women). *, *p* < 0.05.

As additional evidence for the absence of cholesterol-enriched domain loss by vesiculation, patient pEl RBC membrane had even higher cholesterol content compared with 9 healthy donors. In contrast, ceramide levels were slightly decreased, without modification of sphingomyelin species (Figure 4A–C). The decrease of ceramides did result neither from metabolism into sphingosine or sphingosine-1-phosphate nor from ceramide loss by vesiculation (Figure 4D and data not shown). Phosphatidylcholine and PS species, in particular those having long and unsaturated fatty acids, were strongly reduced as compared to a series of healthy donors (Figure 4E,G and Figure S8A–D). Such lipid modifications were not due to the higher reticulocyte content in pEl since cholesterol and sphingomyelin have been shown to instead decrease and phosphatidylcholine and PS species to increase in reticulocytes vs. mature RBCs [32]. They could be related to the decreased content in very long chain fatty acid elongation protein 2 (Figure S6B), but also, in the case of PS, to the strong increase in several monoacyl lysoPS derivatives (Figure 4F,H).

### 3.7. pEI RBCs Displayed Impaired Ca^2+^ Exchange and Increased Ca^2+^ Accumulation, Oxidative Stress, and Hemoglobin Release

**Figure 5:**
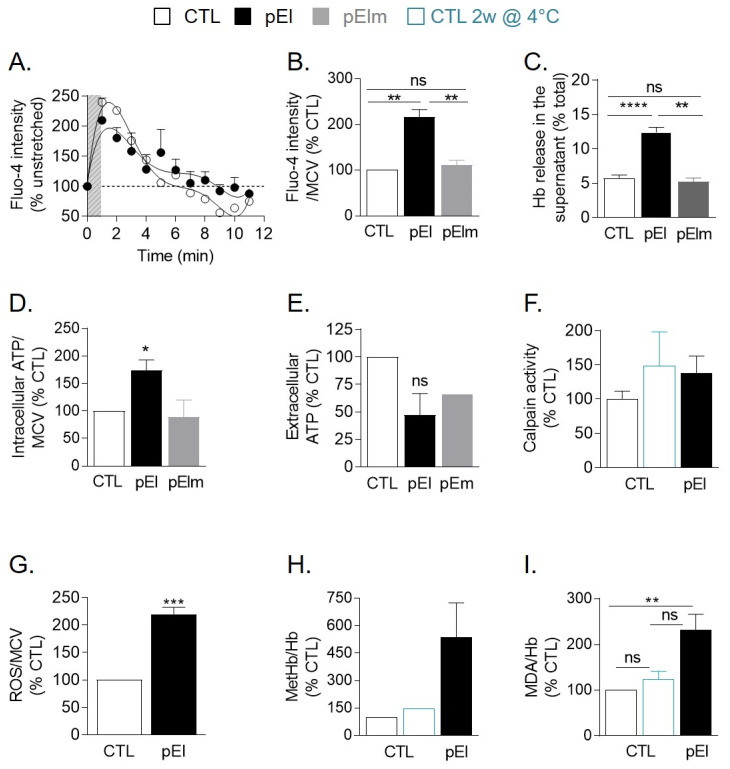
Impaired Ca^2+^ exchange and increased intracellular Ca^2+^ in RBCs from patient pEl were accompanied by increased fragility, ROS accumulation as well as hemoglobin and lipid oxidation. (**A**) Ca^2+^ exchange during RBC deformation in PDMS chambers. RBCs were labeled with Fluo-4 AM and fluorescence intensity was measured before (0 min), during (1 min; grey stripped zone) and after stretching (1–10 min). Data are expressed as percentage of unstretched RBCs and are means ± SEM of 5 experiments where 150–200 RBCs were analyzed at each time. (**B**) RBC intracellular Ca^2+^ content measured by Fluo-4 AM and expressed relative to the mean corpuscular volume (MCV). Data are means ± SEM of 5–10 independent experiments. Kruskal-Wallis test followed by Dunn’s multiple comparisons test. (**C**) RBC fragility evaluated through hemoglobin (Hb) release in isosmotic medium. Data are means ± SEM of 25 independent experiments, except for pElm (3 determinations). Kruskal-Wallis test followed by Dunn’s multiple comparisons test. (**D**,**E**) Intracellular and extracellular ATP contents measured by luminescence after RBC or supernatant incubation with luciferase and luciferin. In D, data are expressed by reference to MCV and are means ± SD/SEM of 2–4 independent experiments. In E, data are means ± SD/SEM of 1–3 independent experimens. Mann-Whitney test. (**F**) Calpain activity determined by calpain activity assay kit (means ± SD of 6 determinations from 2 independent experiments). 2-week-old RBCs were used as positive control. (**G**) Intracellular ROS level measured on washed RBCs incubated with 2′,7′-dichlorodihydrofluorescein diacetate (H_2_DCFDA). Data are means ± SEM of 10 independent experiments. Mann-Whitney test. (**H**) Methemoglobin (metHb) content assessed by a sandwich ELISA. Data were then normalized to the total hemoglobin (Hb) content and are expressed as means ± SD of 3 determinations from 2 independent experiments. (**I**) Membrane lipid peroxidation. Washed and lysed RBCs were measured for malondialdehyde (MDA), a natural product of lipid peroxidation which forms an adduct with thiobarbituric acid (TBA). The adduct was then quantified by fluorimetry. Data are means ± SEM of 3–5 independent experiments. Kruskal-Wallis test followed by Dunn’s multiple comparisons test. ns, not significant; *, *p* < 0.05; **, *p* < 0.01; ***, *p* < 0.001; ****, *p* < 0.0001.

Since GM1- and sphingomyelin-enriched domains were suggested to contribute to Ca^2+^ exchange [10], we then explored the potential consequences of membrane lipid alterations in pEl RBCs for Ca^2+^ exchanges upon RBC deformation in stretchable PDMS chambers [10]. Compared with RBCs from healthy individuals, pEl RBCs showed decreased Ca^2+^ entry together with reduced and delayed exit (Figure 5A). Surprisingly, the intracellular Ca^2+^ content in the resting state was increased by ~2-fold in pEl as compared to a series of healthy donors (Figure 5B and Figure S8E). This modification was accompanied by a similar ~2-fold increase of hemoglobin release (Figure 5C and Figure S1K,L). In contrast, neither Ca^2+^ accumulation nor increased hemoglobin release were observed in pElm (Figure 5B,C).

Mechanistically, altered Ca^2+^ exchange was not due to limited abundance of PIEZO1 and PMCA4 (Figure S6C). Ineffective Ca^2+^ extrusion due to disruption of the ATP pool fueling PMCA was also excluded since intracellular ATP content was even increased in pEl RBCs (Figure 5D). The tendency to decrease of extracellular ATP observed for pEl (Figure 5E) rather suggested impaired activity of PIEZO1, known to not only allow for cation influx but also to regulate mechanotransductive release of ATP from RBCs [35]. Since Ca^2+^ content in pEl RBCs decreased upon Ca_v_ channel inhibition with ω-agatoxin [36] (Figure S9A), this suggested that Ca_v_ channels could take over from PIEZO1.

We then analyzed the potential consequences of Ca^2+^ accumulation in pEl RBCs. The Ca^2+^-dependent calpain activity rose slightly, as in stored RBCs (Figure 5F). The intracellular reactive oxygen species (ROS) content increased by >2-fold (Figure 5G as compared to a series of healthy donors (Figure S8F)). This increase seemed partly Ca^2+^-dependent but did not result from impaired antioxidant defense (Figure S9B,C) and was accompanied by higher methemoglobin content and hemoglobin membrane retention (Figure 5H and Figure S9D,E). Lipid peroxidation also increased, as revealed by the increased generation of malondialdehyde (Figure 5I), a product of polyunsaturated fatty acid oxidation [21], with no evidence for cholesterol oxidation (Figure S9F).

To explore if the oxidative stress could contribute to the pEl phenotype, we used ascorbic acid. This antioxidant restored neither RBC circularity nor resistance to hemolysis, which could be due to its inability to decrease Ca^2+^ content (Figure S10A–D). However, intracellular Ca^2+^ chelation with BAPTA-AM was also unable to restore RBC morphology and hemoglobin release (Figure S10E–G). All those data suggested that the RBC homeostasis in pEl was compromised, as revealed by the increased hemoglobin release and intracellular Ca^2+^ and ROS contents, and that both Ca^2+^ and ROS contributed to the disease.

### 3.8. LysoPS Membrane Insertion in Healthy RBCs Increased Membrane Curvature and GM1-Enriched Domain Abundance and Abrogated Ca^2+^ Exchange

**Figure 6:**
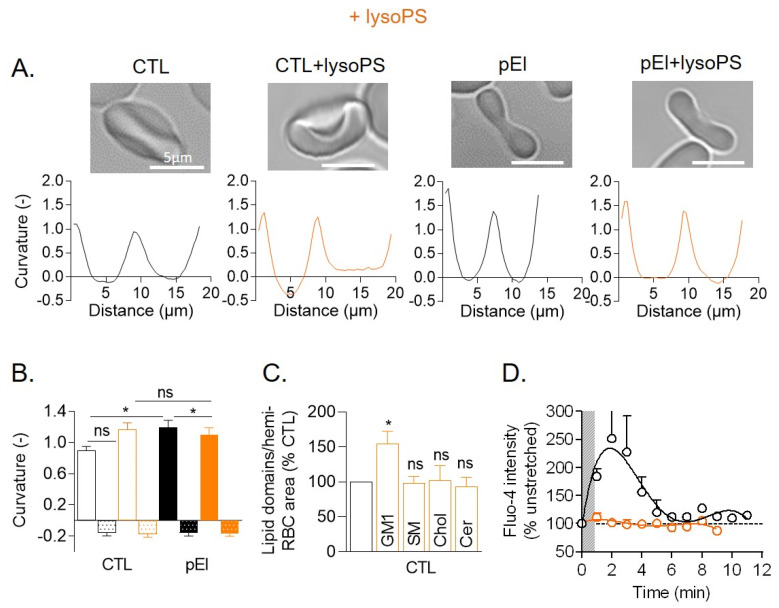
Membrane insertion of lysoPS in healthy RBCs increased membrane curvature and GM1-enriched domain abundance and abrogated Ca^2+^ exchange. RBCs were incubated with or without 18:1 lysoPS and examined for RBC membrane curvature (**A**,**B**), lipid domains (**C**) and Ca^2+^ exchange (**D**). (**A**,**B**) Membrane curvature. RBCs were laid down in IBIDI chambers and analyzed in side view for membrane curvature. The highest and lowest curvatures of each membrane curvature profile were then determined (open and dotted columns, respectively). Data are representative of 4–5 independent experiments with 6–17 RBCs analyzed per experiment. Unpaired t test to compare CTL vs. pEl and paired t test to determine the effect of lysoPS. (**C**) Abundance of lipid domains. RBCs were labeled and visualized by vital fluorescence microscopy as in Figure 3E,F. Data are means ± SEM of 4–11 independent experiments. Paired t tests. (**D**) Ca^2+^ exchange after stretching in a PDMS chamber. The dotted line refers to Fluo-4 intensity of unstretched RBCs. Data are means ± SD of 2 independent experiments. ns, not significant; *, *p* < 0.05.

In the last part of the study, we tried to decipher how membrane molecular and biophysical alterations in pEl could affect RBC morphology and functionality. Intrigued by the pEl RBC resistance to PS surface exposure and by the increased lysoPS content, we started by evaluating whether this cone-shaped lipid could contribute to the disease. To test this hypothesis, lysoPS 18:1 (one of the lysoPS species that increased in the membrane of pEI; see Figure 4H) was inserted into the membranes of RBCs from healthy individuals. As in pEl RBCs, lysoPS insertion increased, although not significantly, membrane curvature in high curvature areas. LysoPS insertion in pEl RBCs instead slightly decreased membrane curvature in high curvature areas. In contrast, no differences between conditions were seen in low curvature areas (Figure 6A,B). LysoPS insertion in healthy RBCs also increased the abundance of GM1-enriched domains and abrogated Ca^2+^ influx upon RBC stretching (Figure 6C,D), as observed in pEl RBCs (see Figure 3E,F and Figure 5A). However in contrast to pEl RBCs, lysoPS membrane insertion did not increase the abundance of sphingomyelin- and ceramide-enriched domains, suggesting additional mechanisms.

### 3.9. Lipid Domain Response to Ca^2+^ Exchange Stimulation Was Impaired in pEl RBCs and Reduction of the Cholesterol Level Worsened the Ca^2+^ Accumulation

**Figure 7:**
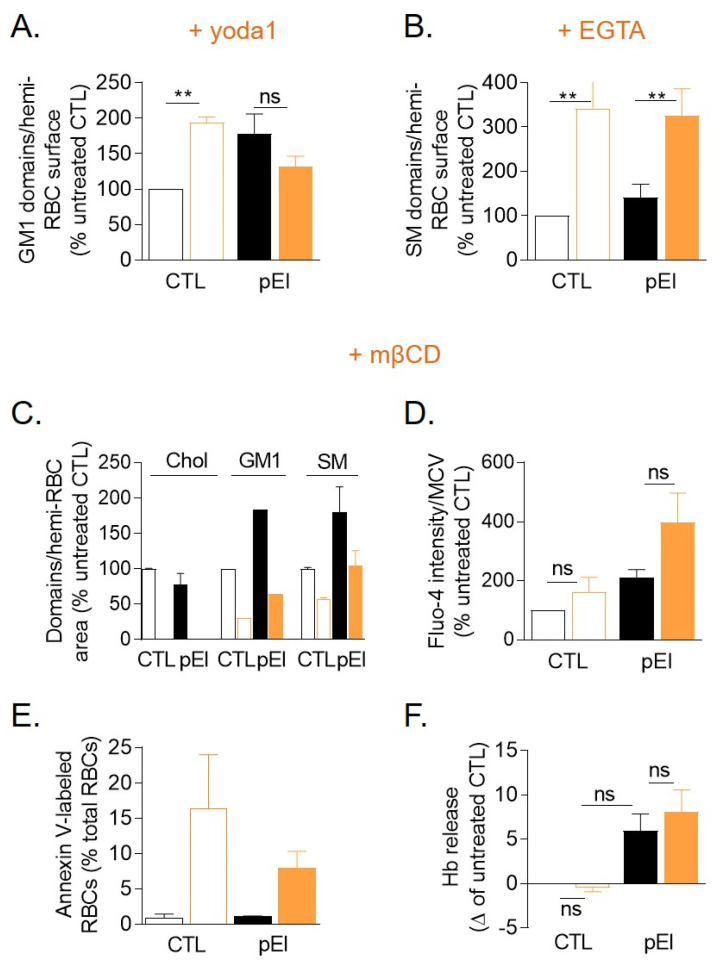
Lipid domains were altered for Ca^2+^ exchange and cholesterol depletion worsened Ca^2+^ accumulation. (**A**,**B**) Abundance of GM1- and sphingomyelin-enriched domains upon Ca^2+^ influx stimulation (**A**, addition of Yoda1 just after labeling) or Ca^2+^ depletion (**B**, incubation with EGTA). Data represent lipid domain abundance per hemi-RBC area and are means ± SEM from 3–7 independent experiments/lipid in which 300–600 RBCs were counted per condition in each experiment. Paired t tests to determine the effect of Yoda1 or EGTA on the abundance of lipid domains of the CTL and pEl. (**C**–**F**) Effect of cholesterol depletion through mβCD of lipid domains (**C**) on Ca^2+^ accumulation (**D**), PS surface exposure (**E**) and hemolysis (**F**). (**C**) Abundance of lipid domains in RBCs pretreated with mβCD. Data are means ± SD/SEM of 2–4 independent experiments. (**D**) Intracellular Ca^2+^ content measured in RBCs incubated with Fluo-4 AM and then with mβCD. Data are means ± SEM of 6 independent experiments. Wilcoxon matched-pairs signed rank tests. (**E**) Surface exposition of phosphatidylserine (PS) evaluated by flow cytometry with Annexin V-FITC on mβCD-treated RBCs. Data are means ± SD of 2 experiments. (**F**) Hemoglobin release in the supernatant measured after incubation with mβCD and expressed as delta of hemolysis detected for the untreated CTL. Data are means ± SEM of 3 independent experiments. Mann-Whitney test for comparison of CTL vs. pEl and Wilcoxon matched-pairs signed rank tests for the effect of mβCD. ns, not significant; **, *p* < 0.01

To address those additional mechanisms, we first evaluated lipid domain functionality by using the PIEZO1 agonist Yoda1 to stimulate Ca^2+^ influx and the extracellular Ca^2+^ chelator EGTA to deplete intracellular Ca^2+^ [10,12]. GM1-enriched domains did not increase upon Yoda1 treatment of pEl RBCs contrasting with a two-fold increase in controls, while sphingomyelin-enriched domains increased by ~2.3-fold upon incubation with EGTA in pEl vs. ~3.5-fold in controls (Figure 7A,B). To examine the potential contribution of the lower domain cholesterol content in such alterations, RBCs were cholesterol-depleted with mβCD (−30% cholesterol [37]) to induce the disappearance of cholesterol-enriched domains and decrease the abundance of GM1- and sphingomyelin-enriched domains (Figure 7C). This treatment still worsened the Ca^2+^ accumulation and slightly increased the RBC fragility in pEl RBCs (Figure 7D,F), suggesting a synergistic effect between the lower cholesterol enrichment of lipid domains in pEl and cholesterol depletion by mβCD. Intriguingly, despite the higher Ca^2+^ content, mβCD-treated pEl RBCs were more resistant to PS surface exposure than healthy RBCs (Figure 7E). Thus, alterations in Ca^2+^ exchange of pEl RBCs could be linked to reduced cholesterol enrichment in lipid domains.

### 3.10. aSMase Inhibition in pEl RBCs Increased Sphingomyelin-Enriched Domains and Partially Restored RBC Morphology and Functionality

**Figure 8:**
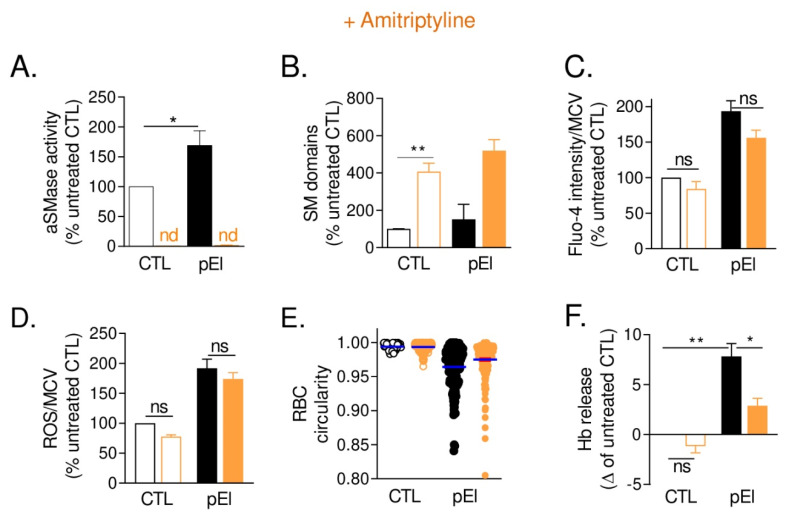
Inhibition of the aSMase activity by amitriptyline in pEl modulated sphingomyelin-enriched domains and partially restored RBC morphology and functionality. Whole blood was incubated with the aSMase inhibitor, amitriptyline (AMI), and assessed for aSMase activity (**A**) while RBCs were washed and tested for sphingomyelin-enriched domain abundance (**B**), Ca^2+^ and ROS accumulation (**C**,**D**), circularity (**E**) and fragility (**F**). (**A**) aSMase activity determined in plasma isolated by Ficoll separation. Data are means ± SEM of 4 independent experiments. Nd, non detectable. Mann-Whitney test. (**B**) Abundance of sphingomyelin-enriched domains determined as in Figure 3E. Data are means ± SD/SEM of 2–4 independent experiments. Paired t test. (**C**,**D**) Intracellular Ca^2+^ and ROS contents measured as in Figure 5B,G. Data are means ± SEM of 3–4 independent experiments. Wilcoxon matched-pairs signed rank tests. (**E**) RBC circularity measured as in Figure S2. One representative experiment out of 4 independent experiments; unpaired t tests: CTL vs. CTL + AMI, *; pEl vs. pEl + AMI, **. (**F**) RBC fragility evaluated in isotonic medium as in Figure 5C. Data are expressed as delta of Hb release by AMI-treated and untreated RBCs (means ± SEM of 6 independent experiments). Mann-Whitney test for comparison of CTL vs. pEl and Wilcoxon matched-pairs signed rank test for the effect of AMI. ns, not significant; *, *p* < 0.05; **, *p* < 0.01.

Since cholesterol can compete for sphingomyelin interaction with ceramide [38], a product of sphingomyelin hydrolysis, we measured the activity of the plasmatic acid sphingomyelinase (aSMase) in pEl and found increased activity (Figure 8A). Upon enzyme inhibition by amitriptyline [39], a four-fold increase in sphingomyelin-enriched domain abundance was observed (Figure 8B). This was accompanied in pEl RBCs by a slight but non-significant reduction of Ca^2+^ accumulation without any effect on ROS content (Figure 8C,D). Hence, RBC circularity and hemoglobin release were significantly improved (Figure 8E,F). Thus, aSMase inhibition by amitriptyline helped to improve the RBC phenotype of pEl.

## 4. Discussion

The patient pEl presents a moderate to severe form of elliptocytosis, resulting from compound heterozygous mutations in the α-spectrin gene SPTA1. The nonsense Arg1811* mutation has not been described before. We showed that expression of the allele carrying this mutation is strongly reduced by nonsense-mediated mRNA decay. The Leu260 Pro mutation, already described [31], is located at the end of the second spectrin repeat, next to the linker between the α2 and α3 repeats, i.e., distal to the tetramerization sites. Although the folding of the domain containing this mutation is normal, the stabilizing interactions between adjacent repeats and stability of the oligomeric protein are disrupted [40]. In a mini-spectrin construct incorporating this mutation, reduced tetramer formation was observed without significant changes in secondary structure [31]. In patients heterozygous for this mutation, like pElm, these effects are well-compensated by the other allele because α-spectrin is produced in large excess compared to β-spectrin [41].

We also documented perturbations of the RBC membrane in pEl. At the molecular level, we found (i) normal spectrin content but its clustering at the RBC edges; (ii) abnormal hemoglobin membrane interaction and its auto-oxidation; (iii) increased oxidized lipids, decreased PS and increased lysoPS species; and (iv) enhanced aSMase activity. At the biophysical level, increased rigidity and curvature and altered lipid domains were observed, while transversal asymmetry was preserved. At the morphological and functional levels, RBC size and circularity, hemoglobin release and Ca^2+^ exchanges through PIEZO1 and PMCA were impaired. From all those alterations, four questions arise.

The first question is whether there is a relation between the cytoskeleton defect and morphology and functionality impairment in pEl RBCs. We indeed observed that the more the variance of the spectrin intensity per RBC increased, the more the RBC size decreased and the hemolysis and intracellular Ca^2+^ content increased (Figure S11A–C). Hence, RBC size and circularity changed proportionally to increased RBC fragility (Figure S11D,E). More importantly, a correlation between the increased Ca^2+^ content and the increased osmotic fragility was observed whether the pEl RBCs were modulated or not for their cholesterol content, oxidative stress and aSMase activity (Figure S11F). This suggested that, as in sickle cell disease, thalassemia or spherocytosis [42], the Ca^2+^ accumulation contributed to the phenotype of pEl. However, the inability of Ca^2+^ chelation by BAPTA-AM to restore RBC morphology and fragility suggested that Ca^2+^ alone was not sufficient to generate the elliptocytotic phenotype and that membrane defects could also contribute to the disease.

The second point therefore relates to the potential link between the cytoskeleton defect and perturbations of membrane biophysical properties and RBC functionality. Several lines of evidence supported the deregulated interplay between the cytoskeleton and lipid domains at the pEl RBC surface and its contribution to RBC fragility. First, the heterogeneous distribution of the cytoskeleton directly impacted lipid domain stability in time and space, as shown for sphingomyelin-enriched domains associated with the RBC low-curvature areas. Second, the enhanced variance of the cytoskeleton intensity per RBC well correlated with the increased GM1- and sphingomyelin-enriched domain abundance at the RBC low-curvature area and with the tendency to decrease of chol-enriched domains at the RBC high-curvature area (Figure S12A). Third, similar correlations were seen with hemoglobin release, suggesting that the deregulated cytoskeleton: domain interplay could contribute to the RBC fragility (Figure S12B). 

The third question is how molecular changes in pEl RBCs can alter the RBC biophysical properties and/or the cytoskeleton elasticity (Figure S13B,C). Among the four membrane biophysical properties measured, i.e., membrane curvature, rigidity, transversal asymmetry, and lateral heterogeneity, only three were modified in pEl RBCs (Figure S13B). The increased membrane curvature in pEl not only resulted from the increased spectrin density at the cell edges but also at least partially from the increased lysoPS content, as shown by mechanistic experiments in healthy RBCs (Figure S13Ba). In pEl RBCs, lysoPS can be generated following activation of the Ca^2+^-dependent NADPH oxidase, which produces oxidized PS intermediates as potential substrates of phospholipase A_2_ [43], and/or the scramblase PLSCR1 [3], which exposes PS to the RBC surface and therefore to plasma phospholipase A_2_ (Figure S13A). Likewise, the decreased ceramide content despite the increased aSMase activity was not due to ceramide metabolism to sphingosine nor to ceramide loss by microvesiculation but could result from ceramide oxidation following flip into the inner leaflet where ceramide-enriched domains were found. The increased membrane rigidity could result from the lower unsaturated-to-saturated phosphatidylcholine ratio and the higher methemoglobin content and lipid peroxidation, but this remains to be proved (Figure S13Bb). Regarding membrane lateral heterogeneity, GM1- and sphingomyelin-enriched domains associated with RBC low curvature areas presented a lower cholesterol content and the latter were also altered by the aSMase activity. In contrast, cholesterol-enriched domains mainly partitioned with the RBC high curvature areas (Figure S13Bc). The preservation of membrane transversal asymmetry in fresh untreated RBCs and the only slight increase of PS surface exposure upon RBC storage and cholesterol depletion was in agreement with the lower membrane PS content, the increase of intracellular ATP content, and thus potentially of the ATP-dependent flippase activity, and the rise of GM1-enriched domain abundance (see below and Figure S13Bd). All those data indicated that molecular defects contributed to alter membrane biophysical properties in pEl RBCs.

Whether this is also the case for the cytoskeleton elasticity can only be speculated since we did not directly measure this elasticity. However, the observation that the bulk membrane lipid order increased in pEl RBCs supported this hypothesis. Cytoskeleton elasticity is known to depend on the intracellular Ca^2+^ and ATP contents as well as on the cytoskeleton density and its anchorage to the membrane. pEl RBCs presented a higher intracellular Ca^2+^ content, which increases the connectivity of the RBC cytoskeleton [44] and results in higher membrane compression [45]. Nevertheless, this more fully connected spectrin network and higher compression can be counteracted by the increased ATP level found in pEl RBCs. The cytoskeleton density was also impaired in pEl RBCs, showing a higher spectrin clustering in high curvature areas and a lower association with low curvature areas. Moreover, reorganization of cytoskeleton:membrane anchorage complexes was revealed by the presence of areas of reduced density alternating with darker areas and by the decreased spatial dissociation between CD47 and glycophorin C, respectively enriched in the ankyrin- and 4.1R- based complexes. Although it remains to determine how altered interactions with anchorage complexes impair elasticity in pEl RBCs, theoretical studies show that the loss of the spectrin:membrane anchorage stiffens the cytoskeleton [46,47]. Hence, the decreased content of PS, a phospholipid normally retained in the inner leaflet and involved in the membrane:cytoskeleton interaction at the 4.1R complexes, and the increased membrane retention of hemoglobin could both contribute to cytoskeleton elasticity impairment. Altogether, our data supported the view that the molecular defects in pEl RBCs, except the increased ATP content, impacted both the RBC biophysical properties and the cytoskeleton elasticity (Figure S13B,C). The increased membrane ghost association of tubulin β6 and components of the protein biosynthesis/folding machinery vs. decreased Rh(D) polypeptide suggested aberrant membrane defects already during erythropoiesis [41,48,49]. Although this remains to be proved, this hypothesis is supported by the fact that membrane rearrangements during erythropoiesis are necessary to generate normal mature RBCs [32].

The fourth key point is how membrane molecular, biophysical, and cytoskeleton elasticity changes can impair RBC functionality in pEl (Figure S13A). Ca^2+^ influx through PIEZO1 was impaired in pEl RBCs, as shown by reduced Ca^2+^ entry upon RBC stretching, despite normal PIEZO1 content (Figure S13A, light grey). Mechanistically, PIEZO1 could be directly impaired by the heterogeneous spectrin lateral distribution at the RBC surface of pEI, as shown upon inhibition of actin polymerization [50]. Moreover, our data pointed to a role for the lower PS and higher lysoPS membrane contents in alteration of PIEZO1 activity through modifications in membrane curvature, as revealed by Ca^2+^ influx abrogation upon stretching of lysoPS-treated RBCs. This is in agreement with the suppression of PIEZO1 activation in myoblasts upon altered PS membrane flip-flop and lysoPS membrane insertion [51]. Besides curvature alteration, lysoPS specifically increased the abundance of GM1-enriched domains, previously suggested to contribute to Ca^2+^ influx through PIEZO1 [10,52]. This hypothesis is supported by the specific excellent correlation between GM1-enriched domain abundance in RBCs at resting state and the Ca^2+^ influx through PIEZO1 upon RBC mechanical activation, revealing that the higher the domain abundance, the lower their ability to induce Ca^2+^ influx (Figure S12C). It remains to be investigated whether there is a spatial and/or functional relationship between PS, lysoPS and GM1-enriched domains. In agreement with this hypothesis, a low number of GM1-enriched domains correlated with an increased PS surface exposure (Figure S12D) and GM1-enriched domains in pEl RBCs did not respond to PIEZO1 activation and presented a lower cholesterol content. Combined with the lower unsaturated-to-saturated ratio of phosphatidylcholine, a phospholipid found to be enriched in those domains [10], these data were in agreement with the cholesterol contribution to PIEZO1 mechanical gating [53] and with the modulation of channel inactivation by long chain polyunsaturated fatty acids [54].

Besides PIEZO1, the PMCA pump activity was also affected in pEI RBCs, as revealed by reduced and delayed Ca^2+^ efflux upon RBC stretching and higher Ca^2+^ accumulation despite normal PMCA content and higher ATP levels (Figure S13A, dark grey). The most obvious mechanism behind could be the oxidative stress, proposed to affect PMCA activity by direct oxidation [55] or through the binding to oxidized calmodulin [56], leading to conformational changes and the formation of aggregates [57]. The implication of those mechanisms in pEl RBCs remains nevertheless to be tested. A non-mutually exclusive alternative mechanism is provided by the alteration of sphingomyelin-enriched domains, previously suggested to participate to Ca^2+^ efflux through PMCA [10,52]. This hypothesis is supported by the specific correlation between sphingomyelin-enriched domain abundance in RBCs at resting state and the extent of Ca^2+^ efflux through PMCA, revealing that the higher the domain abundance, the lower their ability to induce Ca^2+^ efflux through PMCA (Figure S12E). Moreover, sphingomyelin-enriched domains in pEl RBCs exhibited a lower response to intracellular Ca^2+^ depletion by EGTA and increased abundance and rigidity, potentially leading to aSMase activation [13]. It remains to be seen whether the enzyme can modify domain rigidity due to competition between ceramide and cholesterol for sphingomyelin [38], and thereby impair PMCA localization and activity. In support of this hypothesis, PMCA activity in liquid-ordered domains was much higher than PMCA activity excluded from these domains [58]. The relevance of the aSMase-dependent alteration of sphingomyelin-enriched domains for pEl RBCs is provided by two features. First, aSMase inhibition by amitriptyline partly restored the Ca^2+^ content and RBC morphology and resistance to hemolysis. Second, Ca_v_ channel inactivation by agatoxin abrogated Ca^2+^ influx, suggesting the implication of Ca_v_ channels in pEl RBCs, and SMase activity has been shown to stimulate Ca_v_ channels expressed in oocytes [59].

## 5. Conclusions

Our study paves the way towards a better understanding of the molecular mechanism behind elliptocytosis. We demonstrated that aberrant membrane composition and biophysical and cytoskeletal properties affect RBC functionality through Ca^2+^ exchanges in pEl. As a consequence of membrane changes, the RBC homeostasis in pEl is compromised, as revealed by the increased hemoglobin release and intracellular Ca^2+^ and ROS contents. Moreover, the partial restoration of the RBC phenotype upon aSMase inhibition by amitriptyline opens new perspectives for treatment. Although generalization of observations based on only one patient is difficult, the absence of defects in maternal pElm RBCs, the correlations between membrane alterations and RBC morphology and functionality as well as the inability of elliptocytotic RBCs to recover their original biconcave shape due to impaired membrane elasticity [19] strengthen our findings. Hence, altered membrane composition and biophysical properties have also been revealed in a series of patients suffering from spherocytosis, a related cytoskeleton-dependent RBC membrane fragility disease (unpublished data), supporting the importance of membrane:cytoskeleton interplay in RBC morphology and functionality.

## Supplementary Materials

The following figures and supplementary methods are available online at https://www.mdpi.com/2218-273X/10/8/1120/s1. Figure S1: Evolution of diagnostic parameters as well as RBC morphology and fragility in pEl throughout the study, Figure S2: Decreased membrane surface area, perimeter and circularity of pEl RBCs in comparison to pElm RBCs and adult and child healthy RBCs, Figure S3: Gathering of cytoskeleton at one edge of pEl RBCs revealed by transmission electron microscopy and confocal immunolabeling, Figure S4: Altered distribution of membrane proteins from the two anchorage complexes, Figure S5: Lower microvesicle release and slighter RBC morphology modifications upon storage of pEl blood vs. healthy blood, Figure S6: Comparison of pEl and healthy RBCs for ghost-associated proteins involved in protein biosynthesis/folding, lipid metabolism and Ca^2+^ transport/signaling, Figure S7: Lipid domain lateral and transversal membrane distribution and stability in time and space, Figure S8: Comparison of different adult healthy donors for RBC membrane lipid composition and intracellular Ca^2+^ and ROS contents, Figure S9: Ca^2+^ entry in pEl RBCs depends on Ca_v_ channels and the ROS accumulation is partly Ca^2+^-dependent but is not limited by the antioxidant defense and is not accompanied by modification of the oxysterol content, Figure S10: ROS decrease and intracellular Ca^2+^ chelation do restore neither RBC circularity nor resistance to hemolysis, Figure S11: Relation between spectrin heterogeneous distribution and RBC morphology and functionality impairment, Figure S12: Relation between lipid domain abundance and cytoskeleton defect, hemoglobin release, membrane transversal asymmetry and membrane Ca^2+^ exchanges, Figure S13: Model for the effect of molecular changes on RBC membrane functionality, biophysical properties and cytoskeleton elasticity in a moderate to severe case of elliptocytosis.
