# Supplementary material for: Aberrant Membrane Composition and Biophysical Properties Impair Erythrocyte Morphology and Functionality in Elliptocytosis
Source: Biomolecules. 2020 Jul 29;10(8):1120. doi: 10.3390/biom10081120 (PMC7465299; doi:10.3390/biom10081120)
Supplement: Supplementary file 1 [file biomolecules-10-01120-s001.pdf]

biomolecules

Aberrant Membrane Composition and Biophysical Properties Impair Erythrocyte Morphology and Functionality in Elliptocytosis

Hélène Pollet [1,†], Anne-Sophie Cloos [1,†], Amaury Stommen [1,†], Juliette Vanderroost [1], Louise Conrard [1], Adrien Paquot [2], Marine Ghodsi [1], Mélanie Carquin [1], Catherine Léonard [1], Manuel Guthmann [1], Maxime Lingurski [1], Christiane Vermylen [3], Theodore Killian [4], Laurent Gatto [4], Mark Rider [5], Sébastien Pyr dit Ruys [5], Didier Vertommen [5], Miikka Vikkula [6,7], Pascal Brouillard [6], Patrick Van Der Smissen [1], Giulio G. Muccioli [2] and Donatienne Tyteca [1,*]

[1] CELL Unit & PICT imaging Platform, de Duve Institute, UCLouvain, 1200 Brussels, Belgium; hpollet@hotmail.com (H.P.); anne-sophie.cloos@uclouvain.be (A.-S.C.); amaury.stommen@uclouvain.be (A.S.); juliette.vanderroost@uclouvain.be (J.V.); louise.conrard.lc@gmail.com (L.C.); marine.ghodsi@student.uclouvain.be (M.G.); melanie.carquin@gmail.com (M.C.); c.leonard.hellin@gmail.com (C.L.); manuel.guthmann@student.uclouvain.be (M.G.); maxime.lingurski@uclouvain.be (M.L.); patrick.vandersmissen@uclouvain.be (P.V.D.S.)

[2] Bioanalysis and Pharmacology of Bioactive Lipids Research Group, Louvain Drug Research Institute, UCLouvain, 1200 Brussels, Belgium; adrien.paquot@uclouvain.be (A.P.); giulio.muccioli@uclouvain.be (G.G.M.)

[3] PEDI Unit, Institut de Recherche Expérimentale et Clinique & Saint-Luc Hospital, UCLouvain, 1200 Brussels, Belgium; christiane.vermylen@uclouvain.be

[4] Computational Biology and Bioinformatics Unit, de Duve Institute, UCLouvain, 1200 Brussels, Belgium; theodore.killian@uclouvain.be (T.K.); laurent.gatto@uclouvain.be (L.G.)

[5] PHOS Unit & MASSPROT Proteomics Platform, de Duve Institute, UCLouvain, 1200 Brussels, Belgium; mark.rider@uclouvain.be (M.R.); sebastien.pyrditruys@uclouvain.be (S.P.d.R.); didier.vertommen@uclouvain.be (D.V.);

[6] Human Molecular Genetics, de Duve Institute, UCLouvain, 1200 Brussels, Belgium; miikka.vikkula@uclouvain.be (M.V.); pascal.brouillard@uclouvain.be (P.B.)

[7] Walloon Excellence in Life Sciences and Biotechnology (WELBIO), de Duve Institute, UCLouvain, 1200 Brussels, Belgium

* Correspondence: donatienne.tyteca@uclouvain.be

† Authors equally contributed to the work.

Supplemental methods, figure legends and supplemental figures

Supplemental methods

Sequencing. DNAs were extracted from whole blood using Wizard genomic DNA purification kit (Promega). SPTA1 gene was sequenced by Ion Torrent technology using a custom-designed Ampliseq panel (www.ampliseq.com) covering the coding exons and 5bp of flanking introns (= splice sites). DNA libraries for pEl were prepared using Ion AmpliSeq Library Kit according to the manufacturer protocol (Life Technologies) with 10 ng of DNA for each of the two Ampliseq primer pools. Sequencing was performed on a Personal Genome Machine (PGM, Life Technologies), with chip 316. The sequences were aligned to the human reference genome (hg19) with the Ion Torrent Suite Server v5 (Life Technologies) in the form of .bam files. These files were imported in Highlander, a software developed in the DDUV Institute (http://sites.uclouvain.be/highlander/), for variant calling with the embarked Torrent variant Caller v5.2 (Life Technologies), annotation and filtering. RNAs were extracted from whole blood using TRIzol reagent (Invitrogen) and retrotranscribed with moloney murine leukemia virus reverse transcriptase (M-MLV RT; ThermoFisher). For RT-PCR, primers were chosen in exons distant from those carrying the changes of interest (sequences and conditions available on request). Amplicons were purified using the Wizard® SV gel and PCR clean-up system from Promega, and sequenced on an ABI3130xl sequencer with the Big Dye Terminator v3.1 chemistry (Applied Biosystems). Chromatograms were analyzed using CLCbio Main Workbench.

Mass spectrometry. RBCs were lysed with 5 mM hypotonic PBS and ghosts were generated by recircularization of membranes in 20 mM PBS [1]. Ghosts were then solubilized in 50 mM tetraethylammonium bicarbonate (TEAB), pH 7.6, 150 mM NaCl, 1% (*v/v*) IGEPAL (CA-630), 0.1% (*w/v*) SDS, 0.4% (*w/v*) dodecyl-β-maltoside, 0.5% sodium deoxycholate, 50 mM sodium fluoride, 5 mM sodium orthovanadate, 0.5 mM phenylmethylsulfonyl fluoride (PMSF). Samples were then incubated with 5 mM dithiothreitol for 30 min at 55 °C and 20 mM chloroacetamide at 22 °C in the dark for reduction and alkylation, respectively. Detergents were then removed using a detergent removal spin column according to manufacturer's instruction (Thermo Fisher Scientific) to obtain proteins in 100 mM TEAB. Proteins (100 µg) were then precipitated with 10% (*w/v*) trichloroacetic acid (TCA), pellets were washed twice with cold acetone and air dried. After resuspension in 50 µl of 100 mM TEAB, samples were digested overnight at 37 °C with 1% trypsin. Isobaric labelling was performed on 10 µg peptides by Tandem Mass Tag (TMT) and assembled according to manufacturer's instruction (Thermo Scientific). Samples were vacuum dried, dissolved in solvent A (0.1%; *w/v*) trifluoroacetic acid in 3.5% (*v/v*) acetonitrile) and 750 ng of peptide mixture were directly loaded onto reversed-phase pre-column (Acclaim PepMap 100, Thermo Scientific) and eluted in backflush mode. Peptides were separated on a reversed-phase analytical column (Acclaim PepMap RSLC, 0.075 x 250 mm, Thermo Scientific) in a linear gradient of 4-32% (*v/v*) solvent B (0.1%; *v/v*) formic acid in 98% (*v/v*) CH_3CN) for 110 min, 32-60% (*v/v*) solvent B for 10 min, 60-95% (*v/v*) solvent B for 1 min and holding at 95% (*v/v*) solvent B for the last 10 min at a constant flow rate of 300 nl/min on an EASY-nLC 1000 UPLC system. Eluting peptides were subjected to NSI source ionization followed by tandem mass spectrometry (MS/MS) using an Orbitrap Fusion Lumos tribrid mass spectrometer (ThermoFisher Scientific) coupled online to a UPLC system. Intact peptide ions were detected and quantified using the synchronous precursor selection (SPS)-based MS3 scan routine implemented in the Orbitrap Fusion Lumos tribrid instrument. The Orbitrap Fusion Lumos was operated at a positive ion spray voltage of 2100 V and a transfer tube temperature of 275 °C. Briefly, a full scan was performed in the range 375–1500 m/z at a nominal resolution of 120,000 and AGC set to 4×10^5, followed by selection of the most intense ions above an intensity threshold of 5000 for collision-induced dissociation (CID)-MS2 fragmentation in the linear ion trap with 35% normalized collision energy. The isolation width was set to 0.7 m/z with no offset. The top 10 fragment ions for each peptide MS2 were notched out with an isolation width of 2 m/z and co-fragmented to produce MS3 scans analyzed in the Orbitrap at a nominal resolution of 30 000 after higher-energy collision dissociation (HCD) fragmentation at a normalized collision energy of 65%. Raw data files from Orbitrap Fusion Lumos were processed using Proteome Discoverer (version 2.3). MS/MS spectra

were searched against the UniprotKB Human proteome reference database (87 489 total sequences). SEQUEST parameters were specified as: trypsin enzyme, two missed cleavages allowed, minimum peptide length of 6, TMT tags on lysine residues and peptide N-termini (+229.1629 Da) and carbamidomethylation of cysteine residues (+ 57.0214 Da) as fixed modifications and oxidation of methionine residues (+ 15.9949 Da) as a variable modification, precursor mass tolerance of 20 ppm, and a fragment mass tolerance of 0.6 Da. Peptide spectral match (PSM) error rates were determined using the target-decoy strategy coupled to Percolator modeling of true and false matches. Reporter ions were quantified from MS3 scans using an integration tolerance of 20 ppm with the most confident centroid setting. An MS2 spectral assignment false discovery rate (FDR) of less than 1% was achieved. Following spectral assignment, peptides were assembled into proteins and were further filtered based on the combined probabilities of their constituent peptides to a final FDR of 1%. The mass spectrometry proteomics data have been deposited in the ProteomeXchange Consortium (http://proteomecentral.proteomexchange.org) via the PRIDE partner repository with the dataset identifier PXD019059 and 10.6019/PXD019059.

Transmission electron microscopy of the RBC cytoskeleton. Formvar/carbon-coated grids were treated with poly-L-lysine for 15 min at 22 °C and washed extensively (3 times in water and 2 times in medium). Grids were then seeded with washed diluted RBCs for 7 min in medium, rinsed 3 times in medium and permeabilized in 0.5% Triton X-100 for 3 min at RT. Grids were then washed 3 times in medium, fixed for 15 min in 1% (*v/v*) glutaraldehyde in 0.1 M cacodylate, washed in buffer and post-fixated in 1% (*w/v*) OsO_4 in 0.1 M cacodylate for 60 min at 4°C. Grids were again extensively washed (6 times 5 min in 0.1 M cacodylate and 3 times in water) and stained in 1% uranyl acetate for 30 min at RT. Finally, samples were washed (6 times 10 min) in water and overnight air-dried. Samples were then observed in the CM12 electron microscope in transmission mode at 80kV.

Blood smears. A blood drop was spread onto a superfrost^{+} slide. The resulting blood smear was fixed in methanol for 5 min, colored with May-Grunwald for 5 min and with Giemsa for 12 min (both from Merck Millipore) and finally washed with water to favor salt precipitation.

SDS-PAGE and Coomassie Blue staining. RBC ghosts were prepared by hyposmotic hemolysis method at 4 °C [1]. Ghosts were then analyzed by sodium dodecylsufalte 4-15% (*w/v*) polyacrylamide gel electrophoresis (SDS-PAGE; BioRad) and PageBlue™ Protein staining solution (ThermoFisher) following Fairbanks et al. instructions [2]. Quantification of the relative abundance of hemoglobin in PageBlue stained gels was performed using Fiji software.

Isolation and analysis of microvesicles. This was performed as in [3]. Briefly, whole blood maintained for 0, 7 or 14 days at 4 °C was centrifuged at 2000× *g* for 10 min. The plasma was recovered and centrifuged again at 2000× *g* for 10 min. The obtained plasma was diluted in sterile filtered PBS and centrifuged at 20000× *g* for 20 min at 4 °C. The resulting pellet was resuspended in sterile PBS before reiteration of the centrifugation step at 20000 g. The final pellet was resuspended in 1ml sterile PBS. Part of the pellet was fixed and allowed to attach for 8 min onto coverslips pretreated with PLL. Coverslips were then washed, fixed on 1% glutaraldehyde in 0.1 M cacodylate and processed by scanning electron microscopy as for RBCs on filters (see above). The other part of the pellet was kept at -80 °C for determination of the MV size and abundance using a Zetaview® from Particle Metrix.

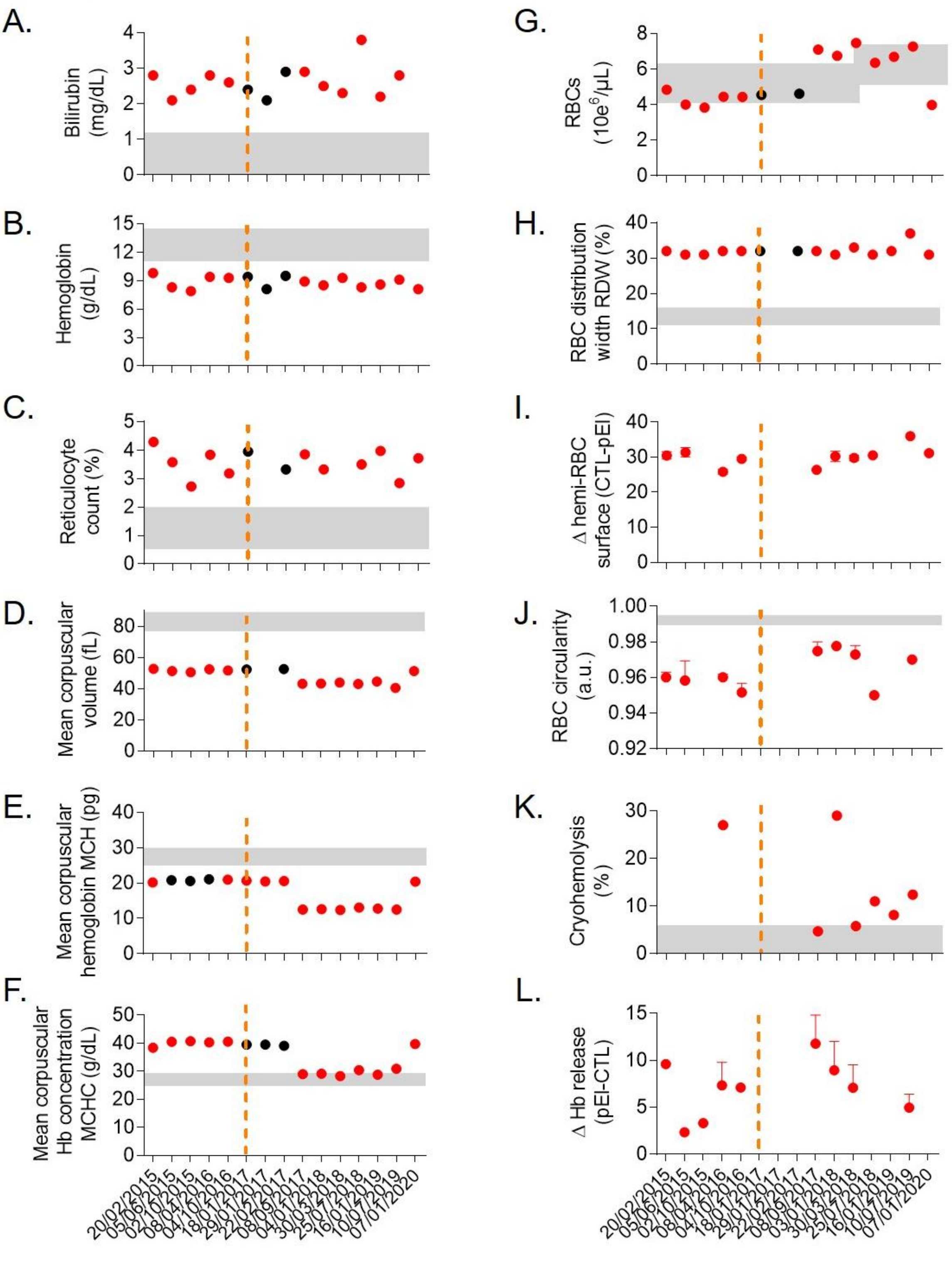

Supplemental Figure 1. Evolution of diagnostic parameters as well as RBC morphology and fragility in pEl throughout the study. (A-H) Parameters used to monitor the disease. Cholecystectomy was performed in January 2017 (orange dotted line) to avoid chronic inflammation due to gallstones. The normal range expected for pEl age is indicated by grey boxes. Red dots indicate venipunctures at which experiments have been performed. **(I-L)** Evolution of RBC surface projection (**I**), circularity (**J**) and fragility (**K**,**L**).

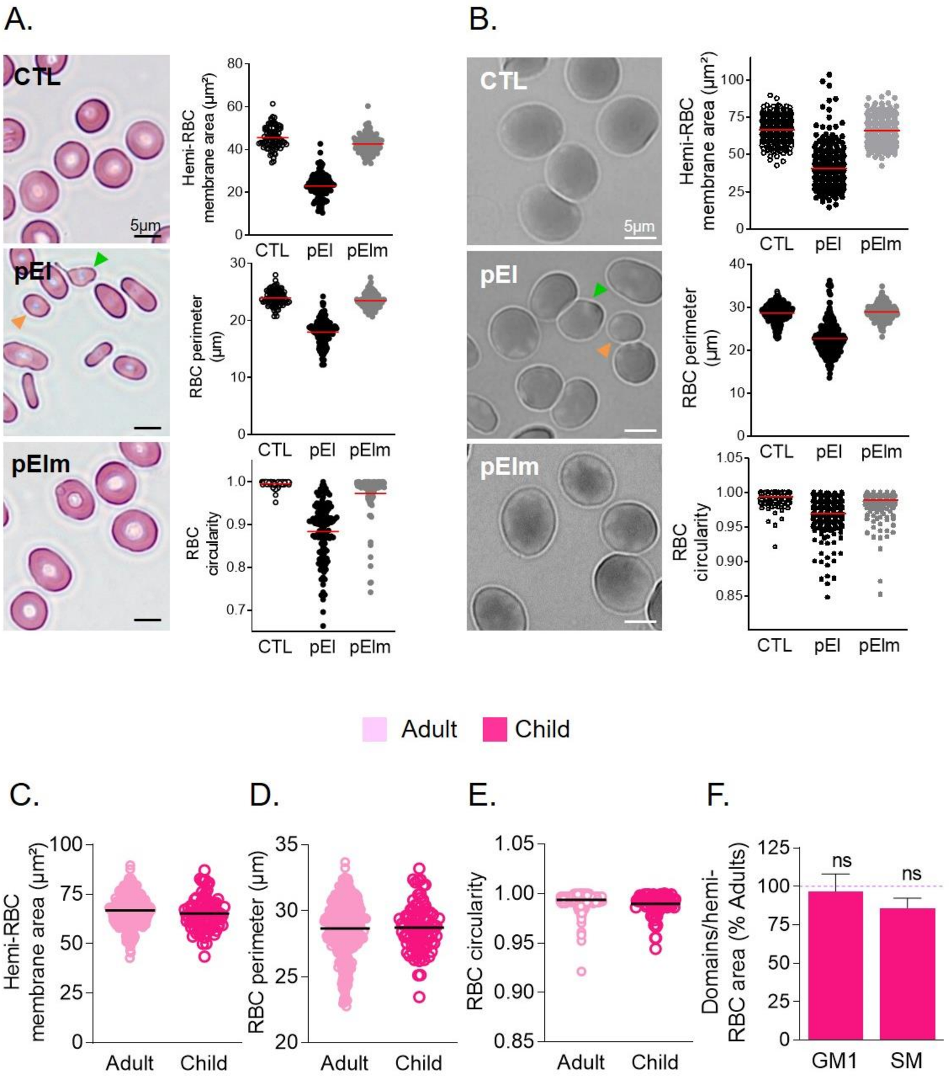

Supplemental Figure 2. Decreased membrane surface area, perimeter and circularity of pEl RBCs in comparison to pElm RBCs and adult and child healthy RBCs. **(A)** Hemi-RBC membrane area, RBC perimeter and circularity determined on May-Grünwald Giemsa-stained blood smears. Left, representative images; green arrowhead, poïkilocytotic RBC; orange arrowhead, small spherical RBC. Right, quantification for one representative blood smear (*n* = 3; Ordinary one-way Anova followed by Turkey's correction test; CTL vs. pEl, ***; CTL vs. pElm, ns; pEl vs. pElm, ***). **(B)** Hemi-RBC membrane area, RBC perimeter and circularity determined on microscopy images of living RBCs laid down on PLL-coated coverslips. Left, representative images; green arrowhead, poïkilocytotic RBC; orange arrowhead, small spherical RBC. Right, quantification shown for one representative PLL-coated coverslip per condition (*n* = 20; Ordinary one-way Anova followed by Turkey's correction test; area and perimeter: CTL vs. pEl: ***, CTL vs. pElm: ns, pEl vs. pElm: ***; circularity: CTL vs. pEl: ***, CTL vs. pElm: *, pEl vs. pElm: *). **(C–F)** Comparison of adult and child healthy donors for RBC morphology and lipid domain abundance. Washed and diluted RBCs from adults or child were spread onto PLL-coated coverslips, labeled with BODIPY-GM1 or -sphingomyelin and directly visualized by fluorescence microscopy. (**C**–**E**) Quantification of the hemi-RBC membrane area, perimeter and circularity for one representative coverslip (*n* = 4; unpaired t test; ns). (**F**) Quantification of lipid domain abundance per hemi-RBC area (means ± SEM from 3 independent experiments/lipid and 300-600 RBCs were counted per condition in each experiment). Unpaired t tests. ns, not significant; *, $p < 0.05$; ***, $p < 0.001$.

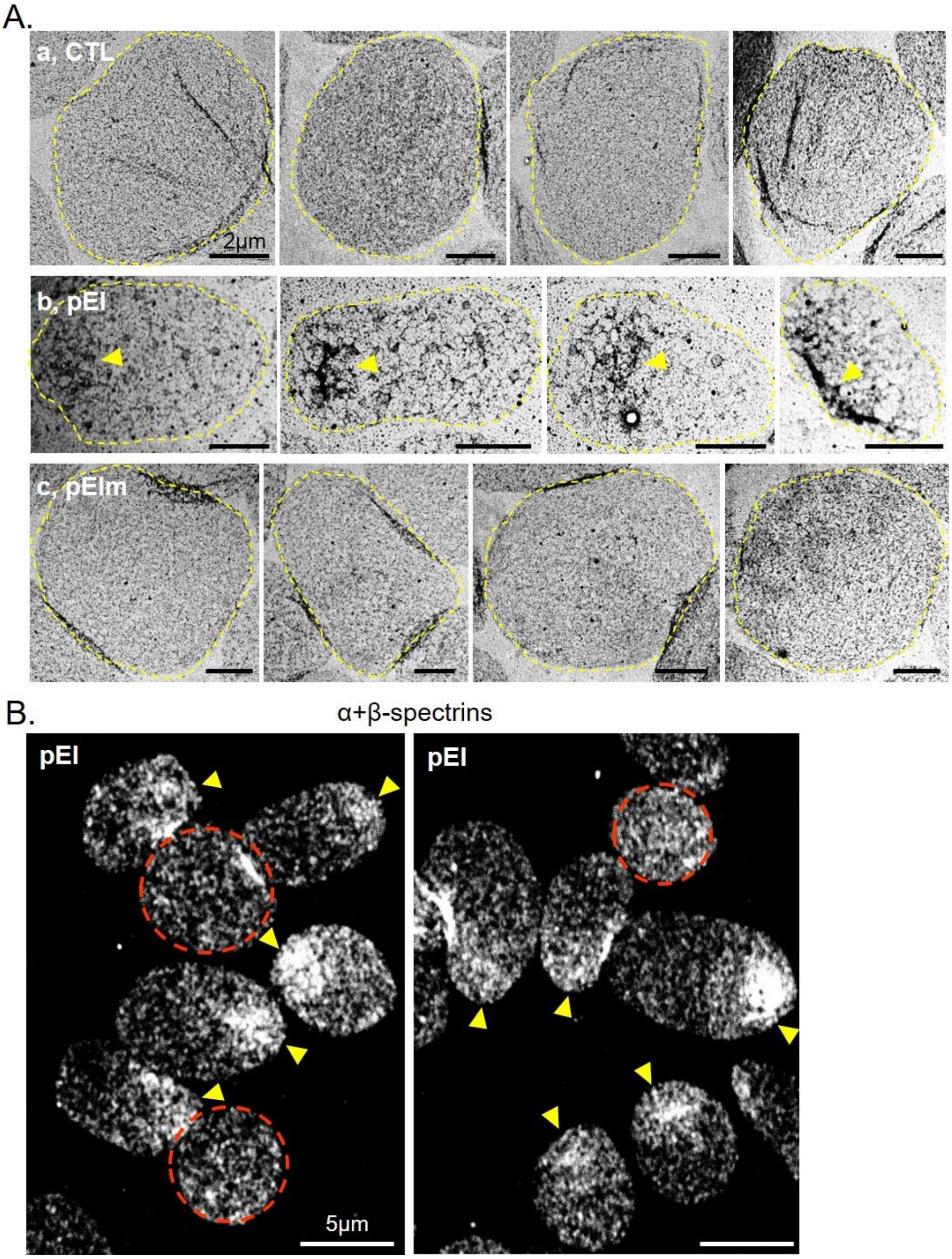

Supplemental Figure 3. Gathering of cytoskeleton at one edge of pEl RBCs revealed by transmission electron microscopy and confocal immunolabeling. (A) RBCs were laid down on PLL-precoated formvar coated grids, permeabilized with 0.5% (*w/v*) Triton X-100, fixed and analyzed by transmission electron microscopy. Yellow lines, RBC outlines. Yellow arrowheads, high local protein density. **(B)** RBCs were laid down on PLL-coated coverslips, permeabilized with 0.5% (*w/v*) Triton X-100, fixed and stained with anti-pan spectrin antibodies. Yellow arrowheads point to increased cytoskeleton density in elliptic RBCs. Red dotted circles highlight circular RBCs.

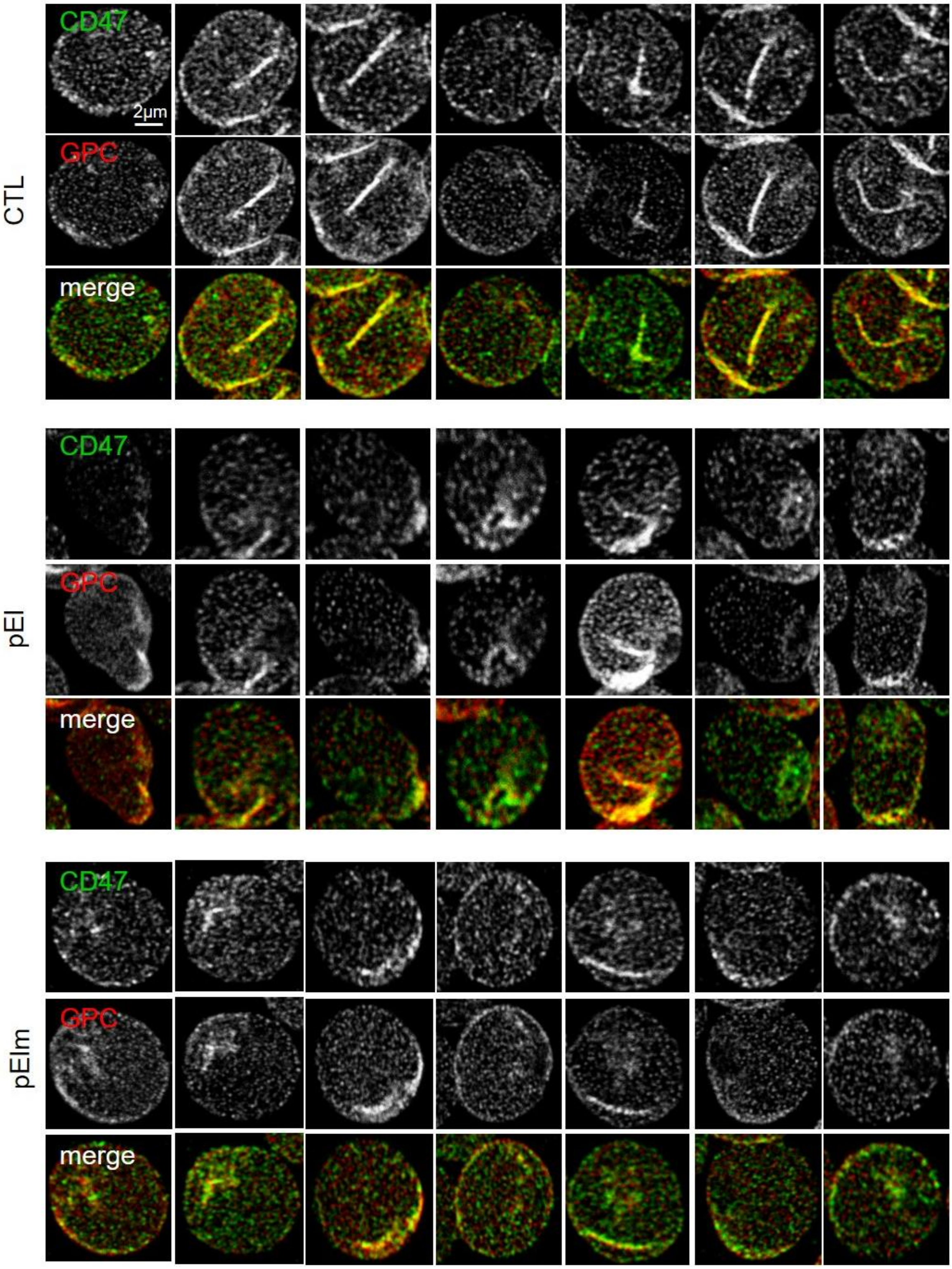

Supplemental Figure 4. Altered distribution of membrane proteins from the two anchorage complexes. RBCs were laid down on PLL-coated coverslips, fixed and stained with antibodies against CD47 (ankyrin complexes; green) and glycophorin C (GPC; 4.1R complexes; red).

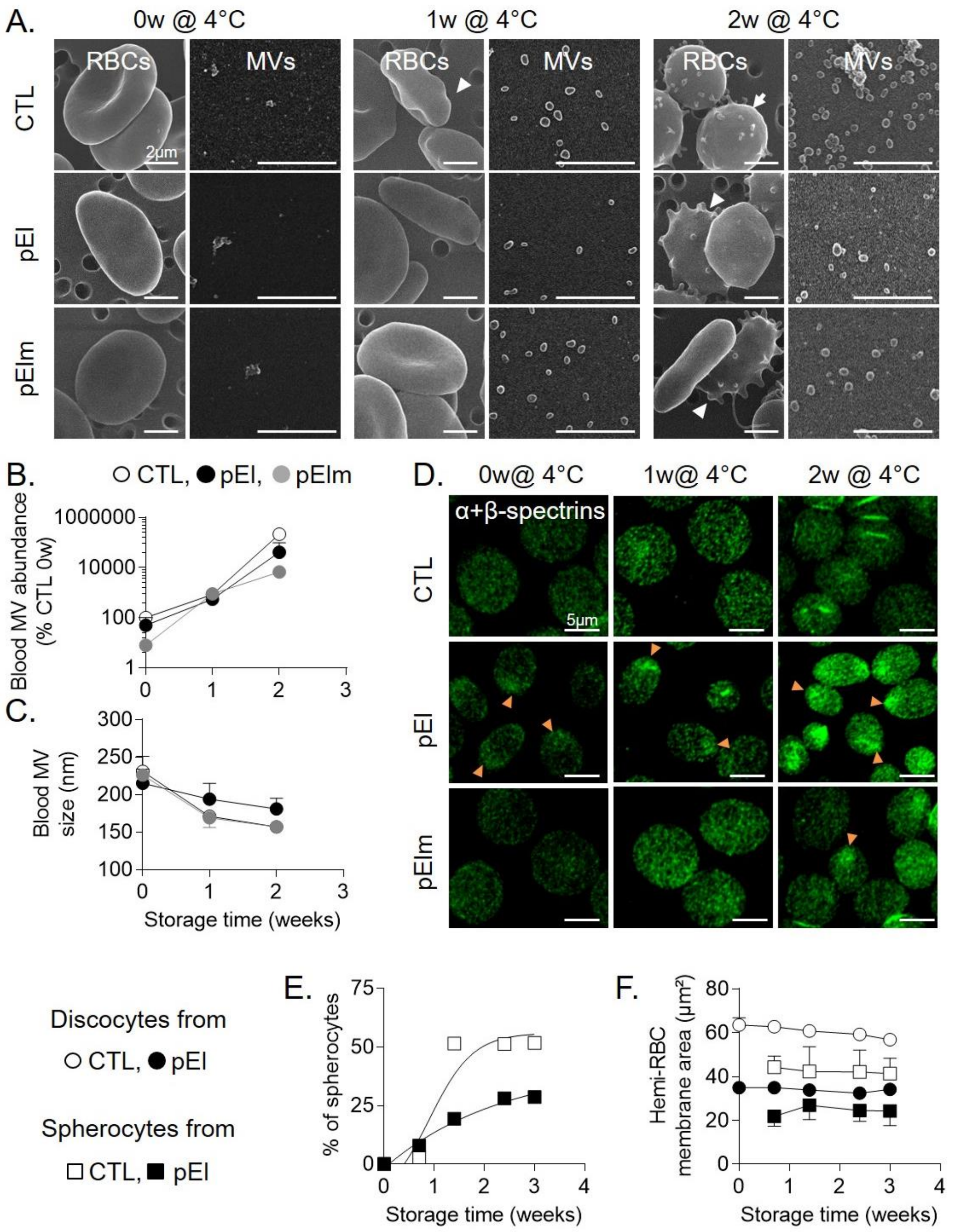

Supplemental Figure 5. Lower microvesicle release and slighter RBC morphology modifications upon storage of pEl blood vs healthy blood. RBCs, either fresh or maintained at 4 °C for the indicated times, were evaluated for morphology, spectrin network, surface area and microvesicle (MV) release. **(A–C)** MV release. (**A**) Representative scanning electron microscopy images. RBCs were washed and fixed in suspension (left images) whereas MVs isolated from those RBCs by multiple centrifugation steps were laid down on PLL-coated coverslips and fixed (right images). Arrowheads, echinocytes; arrows, spherocytes. (**B**) Abundance of MVs (means ± SD of 2 independent experiments). (**C**) Size of MVs (means ± SD of 3 independent measures of the same MV preparations). **(D)** Immunofluorescence of the spectrin network. Data are representative of 3 independent experiments. Orange arrowheads point to increased spectrin density. **(E,F)** Abundance of spherocytes and RBC projected area. RBCs were laid down on PLL-coated coverslips and observed by conventional optical microscopy. The proportion of discocytes vs. spherocytes was counted (**E**) and the projected membrane area was measured with ImageJ software (**F**). Data are means ± SEM of 2-3 independent experiments.

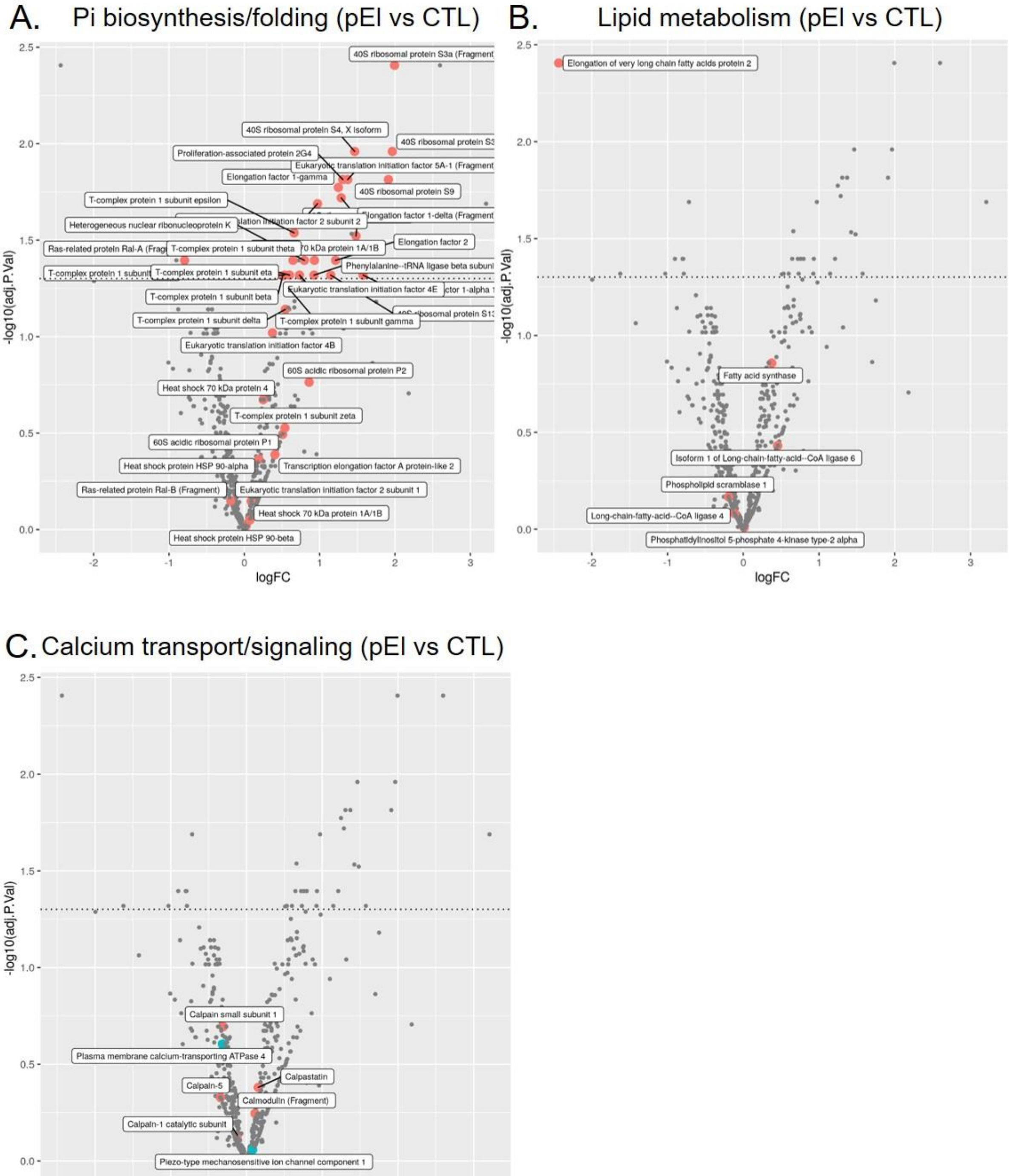

Supplementary Figure 6. Comparison of pEl and healthy RBCs for ghost-associated proteins involved in protein biosynthesis/folding, lipid metabolism and Ca^{2+} transport/signaling. Ghost membranes from pEl and healthy donors were analyzed by differential quantitative mass spectrometry. Volcanoplots show the log_2 fold changes (logFC) in pEl vs healthy donor of 3 independent ghost preparations. Proteins showing a negative or a positive logFC are decreased or increased in pEl vs the healthy donor, respectively. Proteins above the dotted line show a significant difference ($p < 0.05$) in pEl as compared to the control. At panel C, blue symbols, Ca^{2+} transport; red symbols, downstream Ca^{2+} signaling.

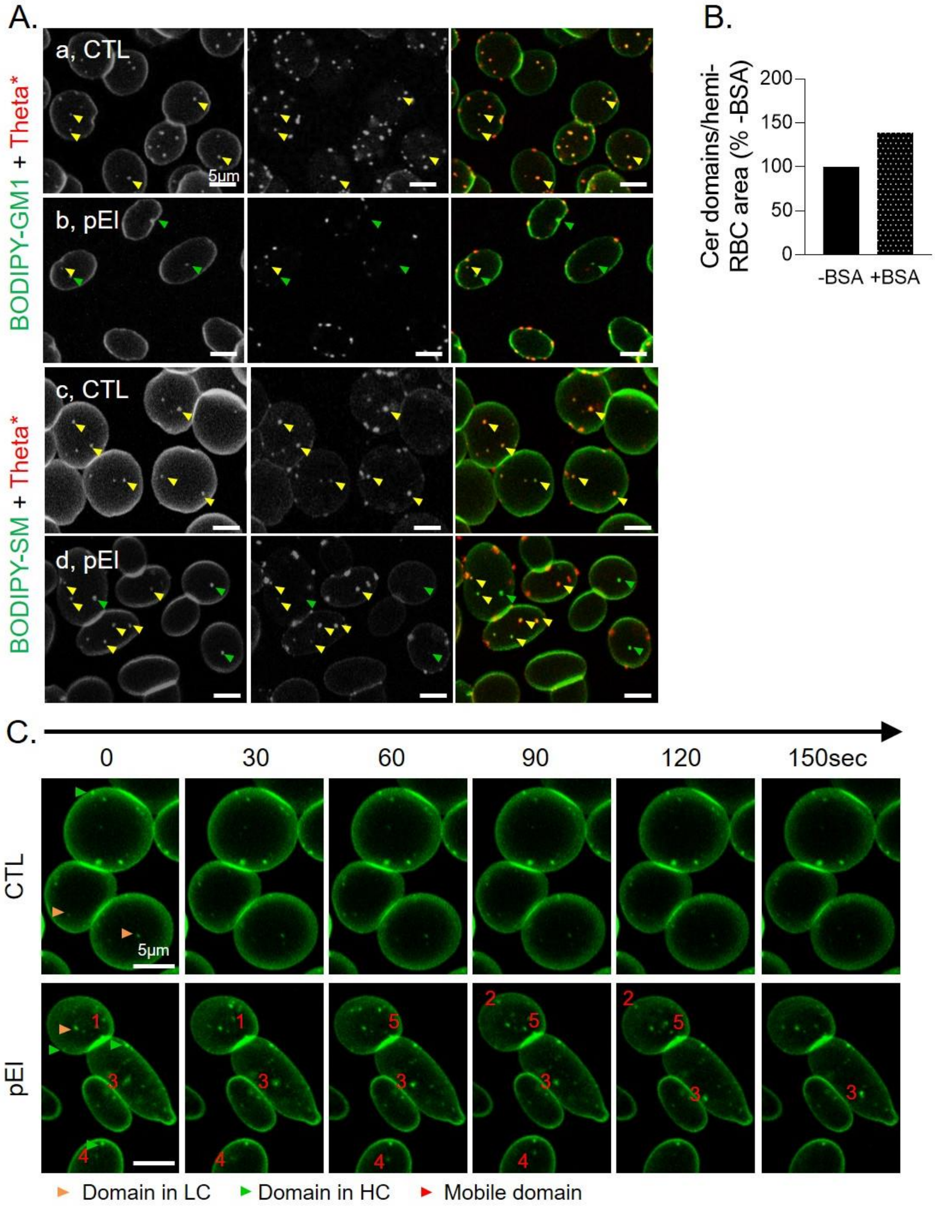

Supplemental Figure 7. Lipid domain lateral and transversal membrane distribution and stability in time and space. (A) Illustration of the lower spatial association of GM1- and sphingomyelin-enriched domains with those enriched in cholesterol in pEl RBCs. RBCs were labeled in suspension with mCherry-Theta (central panels), spread onto coverslips and labeled with either BODIPY-GM1 (a,b) or –sphingomyelin (SM; c,d) (left panels). Green arrowheads, BODIPY-GM1 or -sphingomyelin only; yellow arrowheads, BODIPY-GM1/Theta or BODIPY-sphingomyelin/Theta co-labelled domains. Images presented at (a,b) and (c,d) are representative of 2 and 3 independent experiments, respectively. **(B)** Ceramide-enriched domains in pEl RBCs resist to surface back-exchange by BSA. RBCs were spread onto PLL-coated coverslips, incubated with BODIPY-ceramide and then with 5% BSA or not and directly imaged. Lipid domain abundance per hemi-RBC area was then quantified (means from 1-2 independent experiments in which 120-714 RBCs were counted/condition). **(C)** Sphingomyelin-enriched domains in pEl RBCs are less stable in time and space. RBCs were spread onto coverslips, labeled with BODIPY-sphingomyelin and visualized for 150 sec by confocal microscopy (2 independent experiments). Orange arrowheads, stable domains in low curvature areas (LC); green arrowheads, stable domains in high curvature (HC); red numbers, 'mobile' domains.

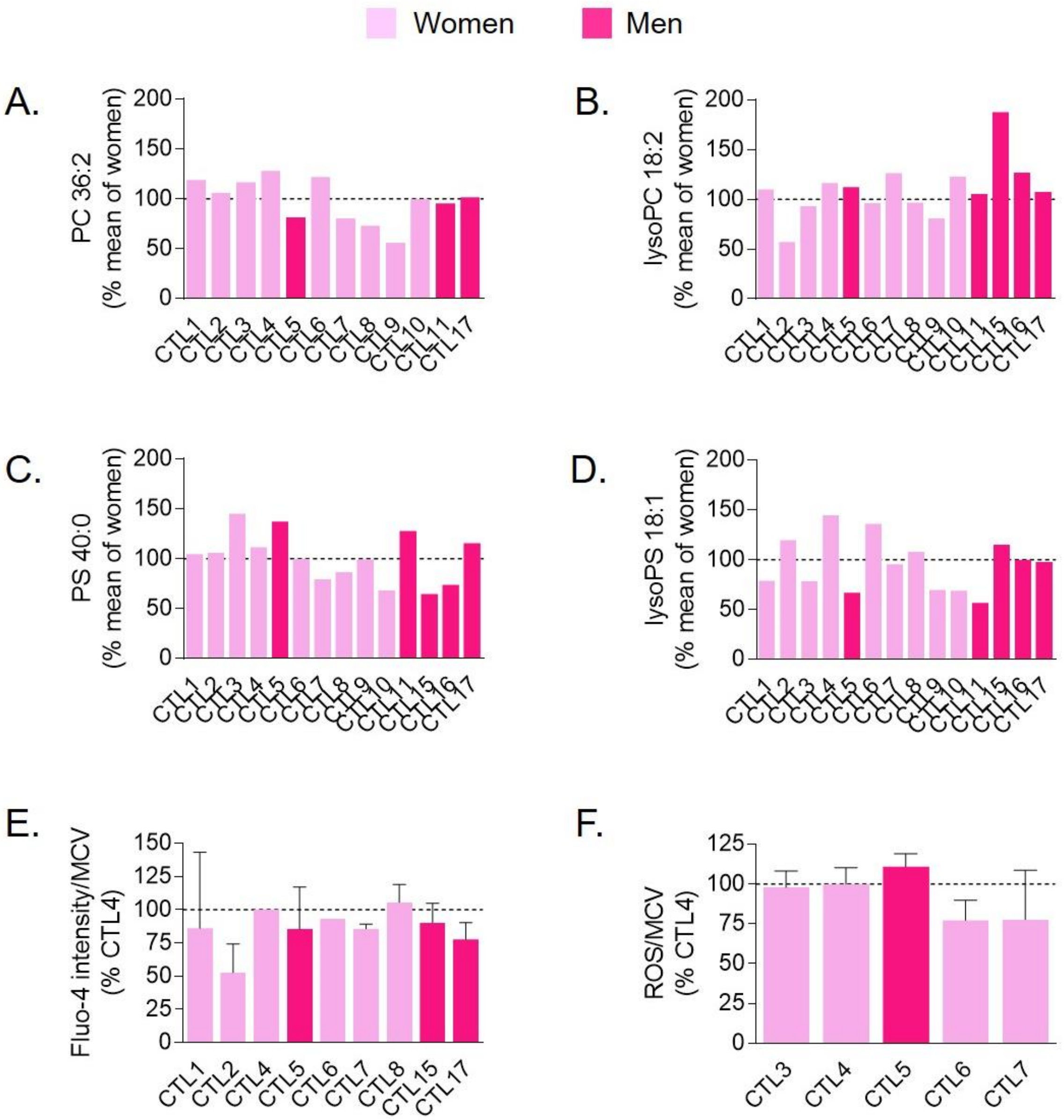

Supplemental Figure 8. Comparison of different adult healthy donors for RBC membrane lipid composition and intracellular Ca^{2+} and ROS contents. (A–D) Content in PC 36:2, lysoPC 18:2, PS 40:0 and lysoPS 18:1 species determined by lipidomics, expressed as percentage of healthy women and taken as examples of lipids highlighted at Figure 4. **(E,F)** Intracellular Ca^{2+} and ROS contents measured as in Figures 5B and G, normalized to MCV and then expressed as percentage of healthy donor 4 (CTL4; means ± SD of 1 to 6 independent experiments).

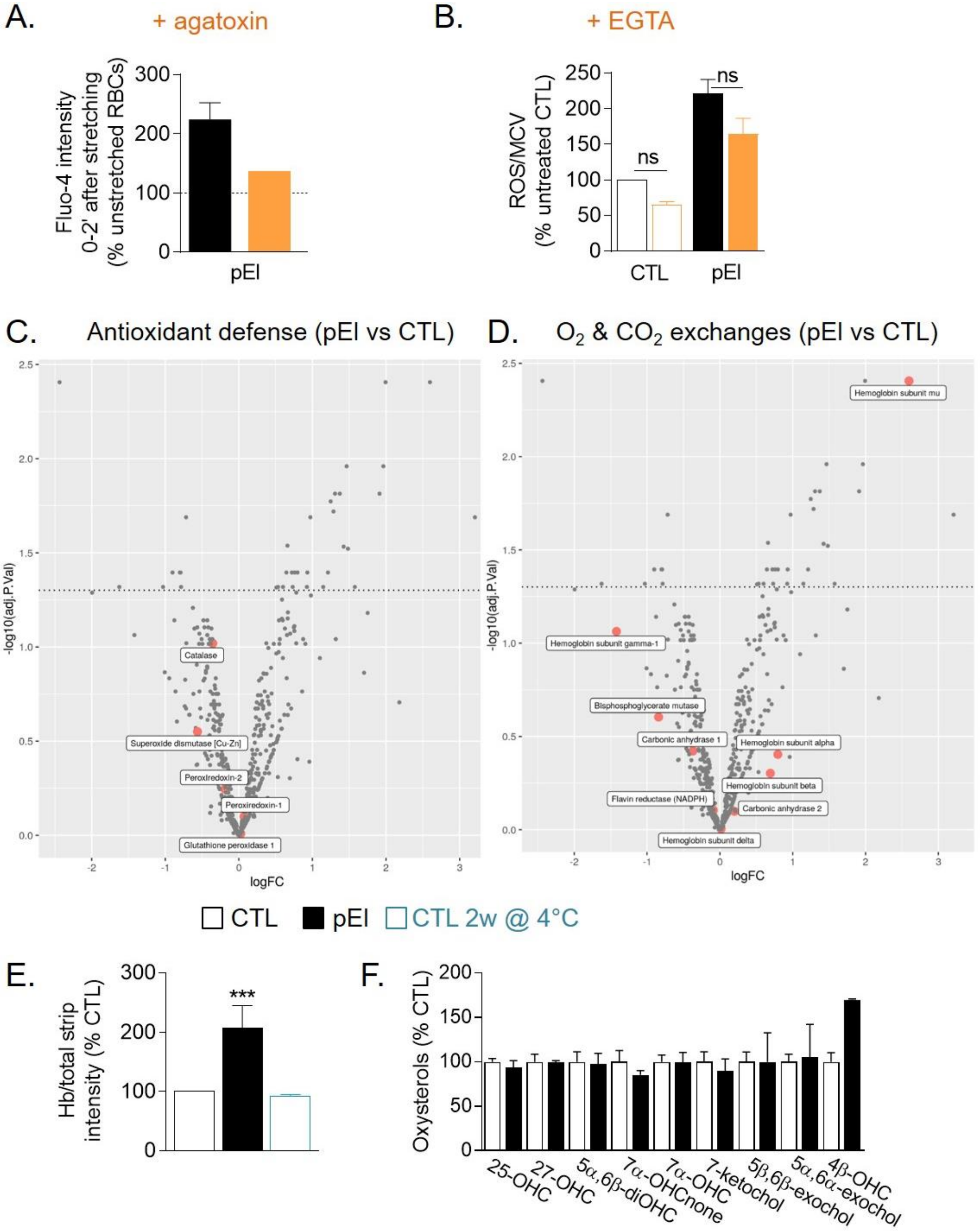

Supplemental Figure 9. Ca^{2+} entry in pEl RBCs depends on Ca_v channels and the ROS accumulation is partly Ca^{2+}-dependent but is not limited by the antioxidant defense and is not accompanied by modification of the oxysterol content. (A) Ca^{2+} entry shortly (0–2 min) after pEl RBC stretching, measured as in Figure 5A, upon Ca_v channels inhibition by ω-agatoxin treatment (1 experiment). **(B)** Intracellular ROS level upon Ca^{2+} depletion with EGTA. Washed RBCs were incubated with 2',7'-dichlorodihydrofluorescein diacetate (H_2DCFDA) in the presence or not of EGTA to deplete intracellular Ca^{2+} (means ± SEM of 3 independent experiments; Wilcoxon matched-pairs signed rank test to compare CTL *vs* CTL EGTA and pEl *vs* pEl EGTA). **(C,D)** Comparison of pEl vs healthy RBCs for antioxidant defense proteins and proteins involved in O_2 and CO_2 exchange. Ghost membranes of pEl and healthy donors were analyzed by relative quantitative mass spectrometry. Volcanoplots show the $\log_2$ fold changes (logFC) in pEl vs healthy donor of 3 independent ghost preparations. Proteins showing a negative or a positive logFC are decreased or increased in pEl vs the healthy donor, respectively. Proteins above the dotted line show a significant difference ($p<0.05$) in pEl as compared to control. **(E)** Hemoglobin membrane association determined by SDS-PAGE and coloration by Coomassie blue (mean ± SEM of 8 independent experiments; Mann-Whitney test). 2 week-old RBCs (blue column) were used as internal comparison (mean ± SD of 2 independent experiments). **(F)** Membrane content in oxysterols. RBCs were washed, lysed, extracted for lipids and determined for (i) tail-oxidized sterols: 25-hydroxycholesterol (25-OHC) and 27-

hydroxycholesterol (27-OHC); and (ii) ring-oxidized sterols: 5α,6β-dihydroxycholesterol (5α,6β-diOHC), 7α-hydroxycholestenone (7α-OHCnone), 7α-hydroxycholesterol (7α-OHC), 7-ketocholesterol (7-ketochol), 5β,6β-epoxycholesterol (5β,6β-exochol), 5α,6α-epoxycholesterol (5α,6α-exochol) and 4β-hydroxycholesterol (4β-OHC). Results are expressed as percentage of control RBCs (mean of 9 healthy women). ns, not significant; ***, $p < 0.001$.

Supplemental Figure 10. ROS decrease and intracellular Ca^{2+} chelation do restore neither RBC circularity nor

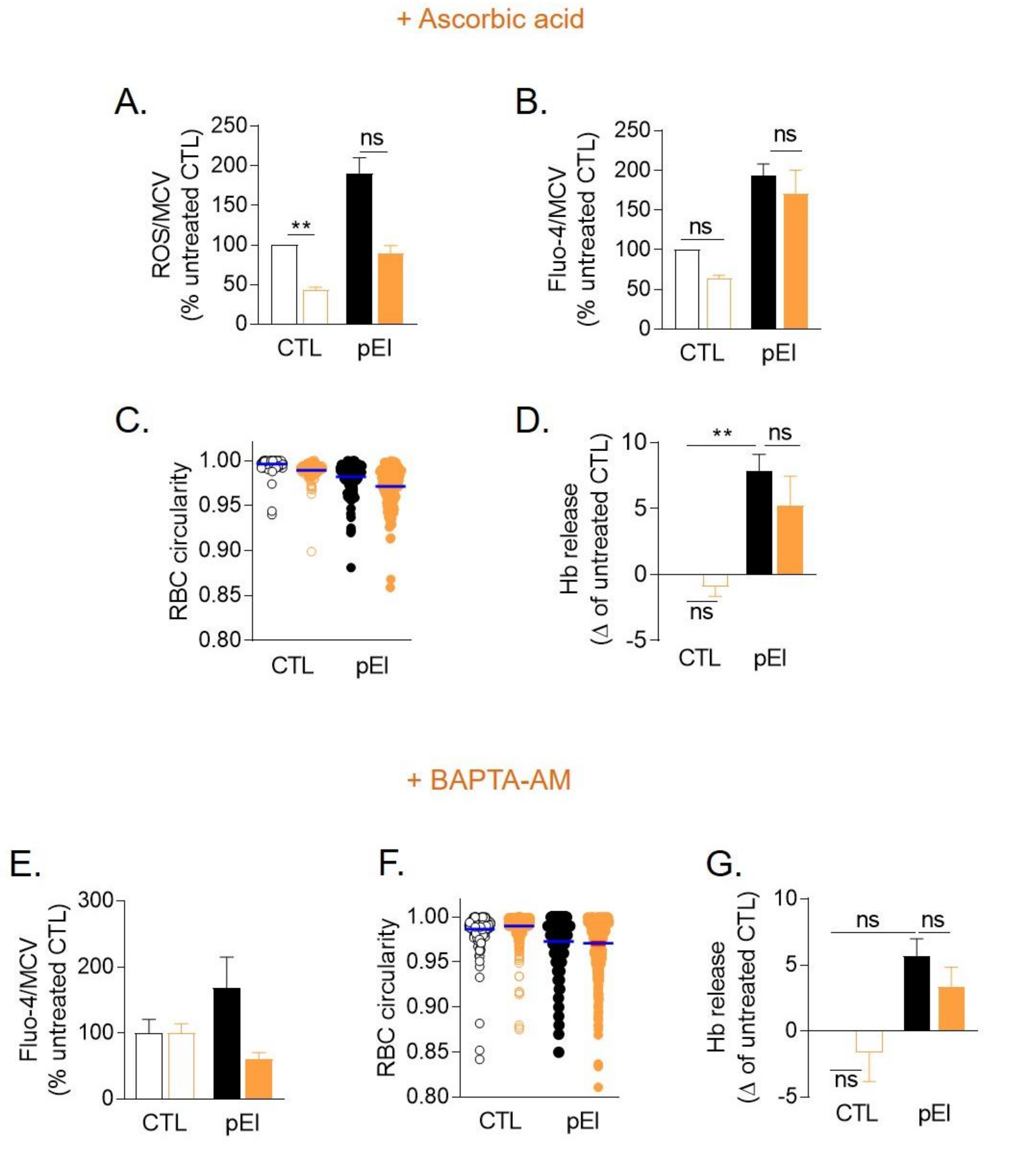

resistance to hemolysis. (A–D) Effect of the antioxidant ascorbic acid (AA). RBCs were incubated with AA, washed and assessed for ROS (**A**), Ca^{2+} (**B**), circularity (**C**) and fragility (**D**)**.** (**A**) Intracellular ROS measured as in Figure 5G (means ± SEM of 3-9 independent experiments; Wilcoxon matched-pairs signed rank test). (**B**) Intracellular Ca^{2+} evaluated as in Figure 5B (means ± SEM of 3 independent experiments; Wilcoxon matched-pairs signed rank test). (**C**) RBC circularity measured as in Figure S2 (one representative out of 4 independent experiments; unpaired t test; CTL vs. CTL+AA, **; pEl *vs* pEl+AA, *). (**D**) RBC fragility evaluated in isotonic medium as in Figure S1L. Data are expressed as delta of Hb release of healthy untreated RBCs (means ± SEM of 6 independent experiments). Mann-Whitney test for comparison of CTL vs. pEl and Wilcoxon matched-pairs signed rank tests for the effect of AA. **(E–G)** Effect of the Ca^{2+} chelator BAPTA-AM. Washed RBCs incubated with BAPTA-AM were washed and assessed for Ca^{2+} (**E**), circularity (**F**) and fragility (**G**)**.** (**E**) Intracellular Ca^{2+} content measured as in Figure 5B (means ± SD of 1 experiment with triplicates). (**F**) RBC circularity measured as in Figure S2 (1 experiment). (**G**) RBC fragility measured in isotonic medium as in Figure S1L. Data are expressed

as delta of Hb release of healthy untreated RBCs (means ± SEM of 3 independent experiments). Mann-Whitney test for comparison of CTL vs. pEl and Wilcoxon matched-pairs signed rank tests for the effect of BAPTA-AM. ns, not significant; **, $p < 0.01$.

Supplemental Figure 11. Relation between spectrin heterogeneous distribution and RBC morphology and

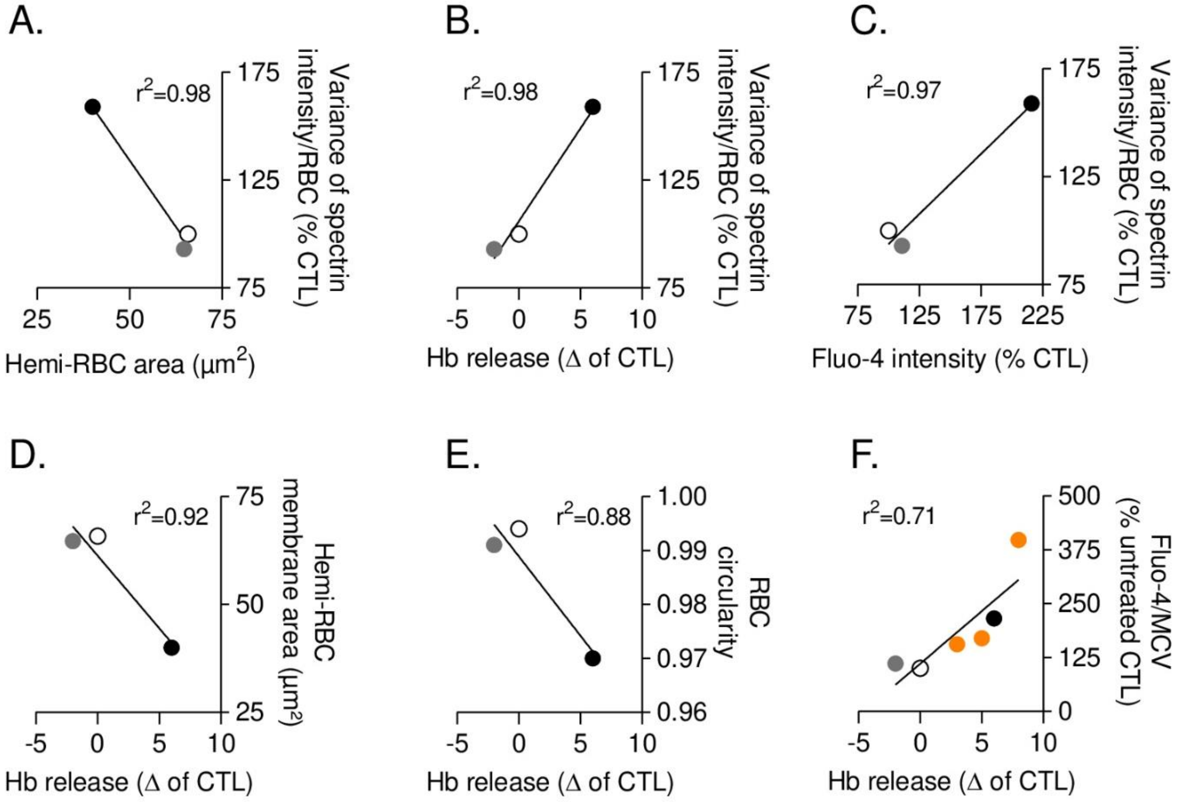

functionality impairment. (A–C) Relation between spectrin heterogeneous distribution and hemi-RBC area determined in Figure S2B (**A**), hemoglobin release determined in Figure 5C (**B**) and Ca^{2+} intracellular content evaluated in Figure 5B (**C**). **(D-F)** Relation between hemoglobin release and hemi-RBC area and RBC circularity measured in Figure S2B (D,E) as well as Ca^{2+} intracellular content determined in Figure 5B, 7D and 8C (**F**). Open symbols, healthy RBCs; black symbols, pEl RBCs; grey symbols, pElm RBCs; orange symbols, pEl RBCs incubated with mβCD, amitriptyline or ascorbic acid.

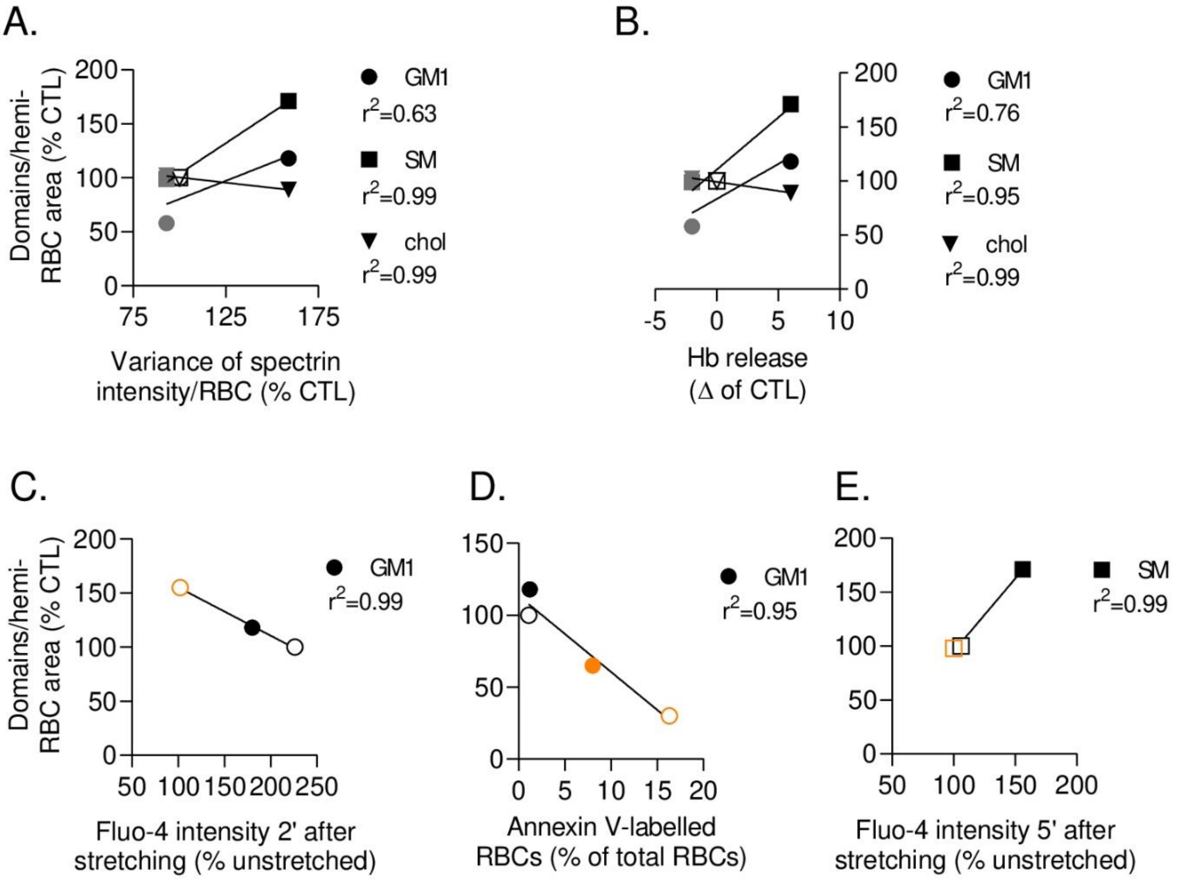

Supplemental Figure 12. Relation between lipid domain abundance and cytoskeleton defect, hemoglobin release, membrane transversal asymmetry and membrane Ca^{2+} exchanges. (A,B) Relation between lipid domains enriched in GM1 (circles), sphingomyelin (squares) and cholesterol (inverted triangles) evaluated in Figure 3F and spectrin heterogeneous distribution determined in Figure 2C (A) or hemolysis assessed in Figure 5C (**B**). **(C)** Relation between GM1-enriched domains determined in Figure 3F and Ca^{2+} influx measured by Fluo-4 intensity 2 min after RBC stretching in Figures 5A and 6D. **(D)** Relation between GM1-enriched domains determined in Figure 3F and PS surface exposure measured in Figure 3C and 7E. **(E)** Relation between sphingomyelin-enriched domains determined in Figure 3F and Ca^{2+} efflux measured by Fluo-4 intensity 5 min after RBC stretching in Figures 5A and 6D. Open symbols, healthy RBCs; black symbols, pEl RBCs; grey symbols, pElm RBCs; orange symbols, treated RBCs.

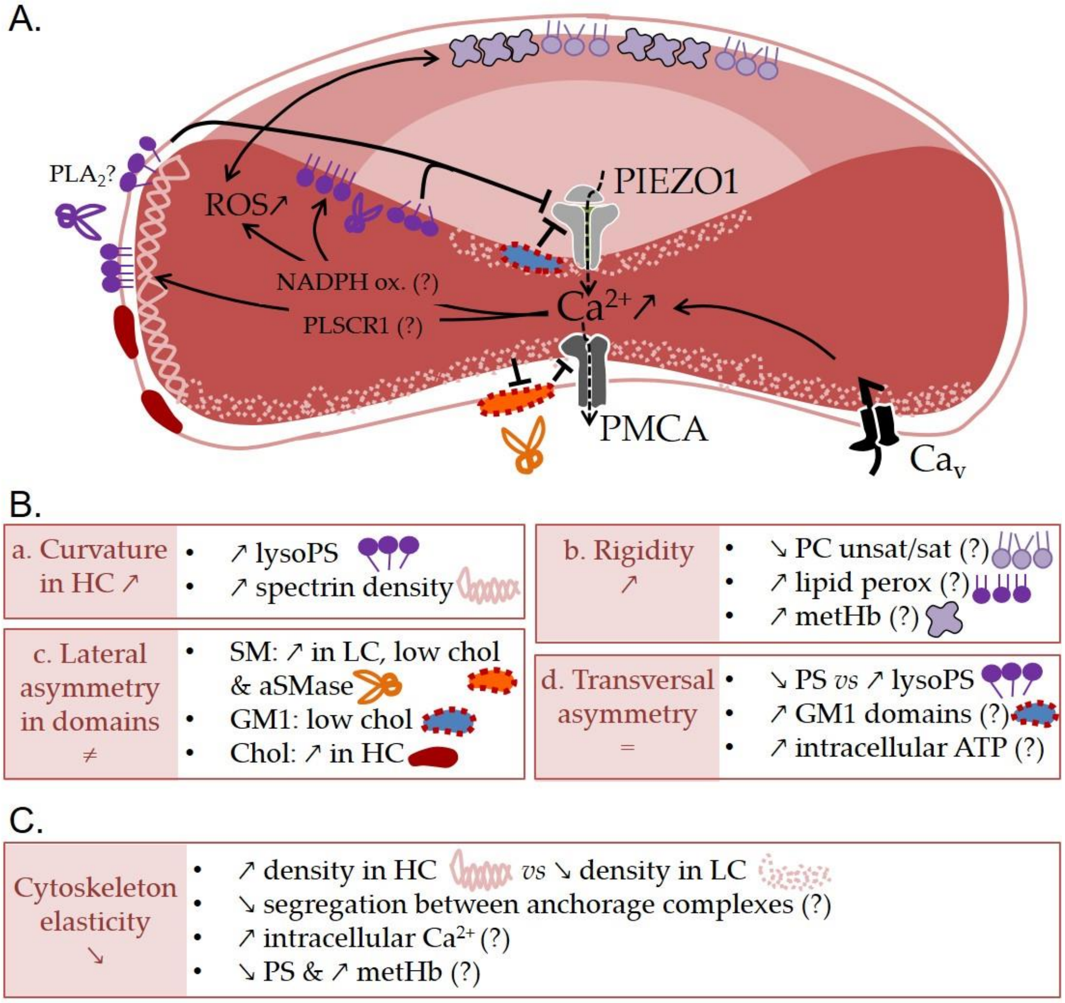

Supplemental Figure 13. Model for the effect of molecular changes on RBC membrane functionality, biophysical properties and cytoskeleton elasticity in a moderate to severe case of elliptocytosis. (A) Effect of molecular changes on membrane Ca^{2+} exchange at the pEl RBC surface. (**B**) Effect of molecular changes on the RBC biophysical properties. (**C**) Effect of molecular changes on the RBC elasticity. (?), remains to be demonstrated. For additional information, see discussion. HC, high curvature; LC, low curvature; PS, phosphatidylserine; lysoPS, lysophosphatidylserine; PC, phosphatidylcholine; SM, sphingomyelin; chol, cholesterol; lipid perox., lipid peroxidation; metHb, methemoglobin; aSMase, plasma acid sphingomyelinase; PLA_2, phospholipase A_2; unsat, unsaturated; sat, saturated.

1. Prausnitz, M.; Lau, B.; Milano, C.; Conner, S.; Langer, R.; Weaver, J. A quantitative study of electroporation showing a plateau in net molecular transport. *Biophys. J.* **1993**, *65*, 414–422, doi:10.1016/s0006-3495(93)81081-6.
2. Fairbanks, G.; Steck, T.L.; Wallach, D.F.H. Electrophoretic analysis of the major polypeptides of the human erythrocyte membrane. *Biochem.* **1971**, *10*, 2606–2617, doi:10.1021/bi00789a030.
3. Cloos, A.-S.; Ghodsi, M.; Stommen, A.; Vanderroost, J.; Dauguet, N.; Pollet, H.; D'Auria, L.; Mignolet, E.; Larondelle, Y.; Terrasi, R.; et al. Interplay Between Plasma Membrane Lipid Alteration, Oxidative Stress and Calcium-Based Mechanism for Extracellular Vesicle Biogenesis From Erythrocytes During Blood Storage. *Front. Physiol.* **2020**, *11*, 712–735, doi:10.3389/fphys.2020.00712.
